# When Powerhouses Fail and Rust Takes Over: Primary Mitochondrial and Lipid Metabolism Failure with Secondary Iron Accumulation and Lipid Peroxidation in Neurodegeneration with Brain Iron Accumulation

**DOI:** 10.1080/17590914.2026.2717255

**Published:** 2026-08-18

**Authors:** Aradhika Vijeev, Shaik Basha, Spandana S. Nadig, Kripa Agarwal, Vibhuti Meharchandani, Aparna Ramakrishna Pai, Krishna Kishore Mahato

**Affiliations:** a Manipal School of Life Sciences, Manipal Academy of Higher Education, Manipal, India; b Department of Biophysics, Manipal School of Life Sciences, Manipal Academy of Higher Education, Manipal, India; c Department of Neurology, Kasturba Medical College-Manipal, Manipal Academy of Higher Education, Manipal, India

**Keywords:** Coenzyme A metabolism, ferroptosis, mitochondrial dysfunction, neurodegeneration with brain iron accumulation (NBIA), precision medicine

## Abstract

Neurodegeneration with brain iron accumulation (NBIA) comprises a genetically heterogeneous group of rare movement disorders characterized by progressive neurodegeneration and selective basal ganglia iron deposition. Recent discoveries have fundamentally reshaped the understanding of NBIA, indicating that defects in coenzyme A metabolism, mitochondrial bioenergetics, lipid remodeling, autophagy–lysosomal pathways, and ferroptosis precede and promote secondary iron dyshomeostasis rather than resulting from primary abnormalities in iron metabolism. This review integrates recent mechanistic and translational evidence (2020–2026) across both common and underrepresented NBIA subtypes, including pantothenate kinase-associated neurodegeneration, phospholipase A2-associated neurodegeneration, COASY protein-associated neurodegeneration, mitochondrial enoyl-CoA reductase protein-associated neurodegeneration, mitochondrial membrane protein-associated neurodegeneration, β-propeller protein-associated neurodegeneration, fatty acid hydroxylase-associated neurodegeneration, neuroferritinopathy, and mitochondrial DNA-associated forms. Unlike previous reviews, this synthesis consolidates findings from patient-derived induced pluripotent stem cell neuronal and glial models, compartment-specific iron localization, advanced neuroimaging biomarkers, and emerging therapeutic strategies within a unified mechanistic framework. Collectively, the evidence supports a paradigm in which mitochondrial dysfunction and lipid metabolic failure initiate disease progression, whereas iron accumulation amplifies oxidative injury and lipid peroxidation, thereby increasing ferroptotic cell death and neuronal degeneration, providing an updated foundation for biomarker discovery, mechanistically informed therapeutic development, and precision medicine approaches in NBIA.

## Graphical Abstract

**Figure:**
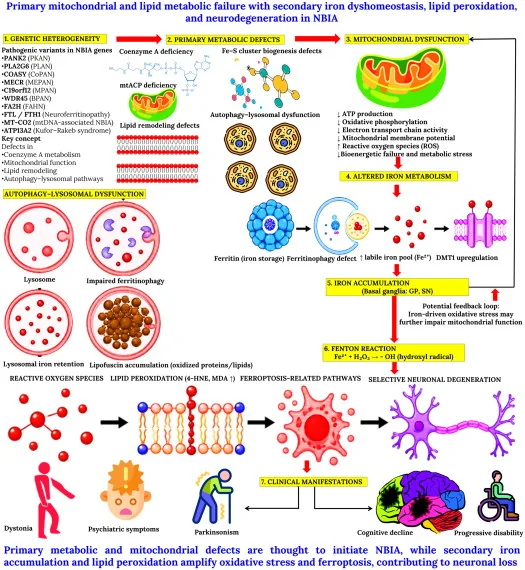

## Introduction

Neurodegeneration with brain iron accumulation (NBIA) is a genetically and clinically heterogeneous group of rare, progressive neurodegenerative movement disorders unified by a shared neuroradiological hallmark: abnormal iron (Fe) deposition within the globus pallidus (GP) and substantia nigra (SN), typically detected as T2-hypointensity on magnetic resonance imaging (MRI) (J.-H. Lee et al., 2020). To date, at least 10–15 monogenic disease entities have been identified, with pantothenate kinase-associated neurodegeneration (PKAN), PLA2G6-associated neurodegeneration (PLAN), mitochondrial membrane protein-associated neurodegeneration (MPAN), and beta-propeller protein-associated neurodegeneration (BPAN) constituting the most common and best-characterized subtypes. Rarer, mechanistically informative forms include COASY protein-associated neurodegeneration (CoPAN), fatty acid hydroxylase-associated neurodegeneration (FAHN), mitochondrial-enoyl-CoA-reductase-associated neurodegeneration (MEPAN), neuroferritinopathy (NFT), and a mitochondrially encoded form associated with cytochrome c oxidase subunit II (MT-CO2) variants (Hayflick, 2023). Iterative gene discovery, historically enabled by international collaborative infrastructures such as the Treat Iron-Related Childhood-Onset Neurodegeneration (TIRCON) network, progressively replaced the eponymous “Hallervorden-Spatz syndrome” designation with a mechanistically defined nosology, and continues to refine the boundaries between core NBIA disorders and phenotypically overlapping conditions such as Kufor-Rakeb syndrome (KRS), which shares basal ganglia iron deposition and Parkinsonian features but arises from a distinct lysosomal pathomechanism (Hayflick, 2023; Karin et al., 2021).

Although Fe accumulation remains the unifying radiological signature of NBIA, accumulating mechanistic evidence indicates that the majority of NBIA-associated genes do not directly encode iron transporters or iron-handling proteins; rather, pathogenic variants converge on coenzyme A (CoA) biosynthesis, mitochondrial bioenergetics and iron-sulfur cluster assembly, membrane lipid metabolism, and autophagic-lysosomal turnover, with secondary iron dyshomeostasis and lipid peroxidation-driven ferroptosis emerging as amplifying, rather than strictly initiating, events in the neurodegenerative cascade (Agostini et al., 2024; Cascone et al., 2026). Despite this conceptual reframing, much of the antecedent NBIA literature has relied disproportionately on findings generated before the widespread adoption of human-induced pluripotent stem cell (iPSC)-derived disease modeling, underrepresented less common but mechanistically informative subtypes such as CoPAN and MEPAN, and inconsistently situated ATP13A2-related KRS relative to the canonical NBIA disease group. Whole-brain iron-sensitive MRI techniques, including quantitative susceptibility mapping (QSM) and R2 relaxometry, together with *ex vivo* analytical methods capable of resolving cell-type- and organelle-specific metal content, have substantially advanced understanding of where iron accumulates, whether within lipofuscin, ferritin, mitochondria, or lysosomes, yet this spatial and compartmental complexity has rarely been synthesized across NBIA subtypes in a single mechanistic framework (Dusek et al., 2022). Concurrently, a substantial and rapidly growing body of work employing human iPSC-derived neurons, astrocytes, and microglia, arguably the most disease-relevant cellular platforms for dissecting cell-type-specific iron handling, mitochondrial failure, endosomal-lysosomal trafficking defects, and neuroinflammatory signaling in PKAN, BPAN, MPAN, and CoPAN- has not been comprehensively integrated into prior syntheses of the field (Agostini et al., 2024).

The present review addresses these unresolved gaps by systematically integrating findings from recent research studies published predominantly within the past five years alongside foundational and recent mechanistic reviews, thereby updating rather than restating the established consensus on NBIA pathobiology (Dusek et al., 2022; Wiśniewska et al., 2025). Its principal contribution is threefold. First, it provides comprehensive coverage of both major and minor NBIA subtypes, PKAN, CoPAN, MEPAN, PLAN, BPAN, MPAN, neuroferritinopathy, and FAHN, organized within a unified pathway-based framework centered on CoA and mitochondrial metabolism, lipid peroxidation, and autophagy/ferroptosis, rather than the traditional iron-centric classification scheme (Wiśniewska et al., 2025). Second, it consolidates human iPSC-derived neuronal, astrocytic, and microglial model data across multiple NBIA subtypes, capturing cell-type-specific phenotypes such as mitochondrial iron deficiency driving cytosolic iron overload, impaired endosomal trafficking, and aberrant immunomodulatory signaling, none of which have been jointly discussed in comparable prior reviews (Agostini et al., 2024; Cascone et al., 2026). Third, it explicitly delineates the nosological relationship between ATP13A2-related KRS and the core NBIA disease group, clarifying a classification ambiguity present in earlier syntheses (Hayflick, 2023; Karin et al., 2021). This positions the present work as distinct from earlier reviews such as those addressing MRI pattern recognition or emerging disease-modifying therapies in isolation, which, while foundational, predate the majority of iPSC-based mechanistic and translational data now available (Cascone et al., 2026; Iankova et al., 2021; J.-H. Lee et al., 2020).

The specific problems this review aims to resolve include fragmentary and outdated coverage of CoA and mitochondrial pathobiology across PKAN, CoPAN, and MEPAN; insufficient mechanistic integration of lipid peroxidation, autophagy, and ferroptosis linking PLAN, BPAN, and MT-CO2-associated disease; limited discussion of the subcellular localization of accumulated iron (lipofuscin, ferritin, mitochondria, or lysosome-associated) and its relationship to neurotransmitter metabolism and imaging biomarkers in the GP; and a therapeutic landscape that has, in prior reviews, lagged behind recent clinical trial and translational advances (Iankova et al., 2021). These problems are systematically investigated across dedicated sections addressing NBIA genetics and epidemiology, CoA and mitochondrial metabolism, lipid peroxidation/autophagy/ferroptosis mechanisms, subtypes beyond PKAN and PLAN, human cellular and iPSC-derived models, iron localization and neurotransmitter/imaging biomarkers, and clinical course and emerging therapies. By explicitly distinguishing causal from consequential roles of iron within the mitochondria-lipid-autophagy axis and anchoring each mechanistic section in recent primary data rather than aggregated older literature, this review aims to provide a current, mechanistically coherent, and cellular-model-inclusive reference for the NBIA research field.

## Methodology

### Literature Search and Selection

A structured, non-systematic narrative review approach was adopted, combining targeted database searches. Searches were conducted across PubMed/MEDLINE, Scopus, Web of Science, and Google Scholar, supplemented by manual screening of reference lists from recently published reviews and primary studies to identify additional eligible articles, including the core reviews retained from the original manuscript and newly incorporated primary research articles.

### Keywords Used

Search terms combined disease-level and gene-level keywords, including “neurodegeneration with brain iron accumulation,” “NBIA,” “PKAN,” “PANK2,” “PLAN,” “PLA2G6,” “BPAN,” “WDR45,” “MPAN,” “C19orf12,” “CoPAN,” “COASY,” “MEPAN,” “MECR,” “FAHN,” “FA2H,” “neuroferritinopathy,” “FTH1,” “MT-CO2,” “Kufor-Rakeb syndrome,” “ATP13A2,” combined via Boolean operators with terms such as “iron accumulation,” “ferroptosis,” “lipid peroxidation,” “mitochondrial dysfunction,” “autophagy,” “iPSC-derived neurons,” “astrocytes,” “microglia,” “iron chelation,” and “clinical trial.”

### Time Frame

The primary search window spanned January 2020 to July 2026, capturing the most recent mechanistic and translational advances along with a few foundational works, particularly iPSC-based cellular modeling studies and clinical trial data.

### Language

Only articles published in English were included, consistent with the predominant language of peer-reviewed biomedical literature in this field.

### Inclusion Criteria

Studies were included if they reported original genetic, molecular, cellular, imaging, clinical, or therapeutic data on NBIA disorders or closely related iron-associated neurodegenerative conditions; were published in peer-reviewed journals; and provided sufficient methodological detail to allow critical appraisal. Both primary research articles and narrative/systematic reviews meeting these criteria were eligible.

### Exclusion Criteria

Conference abstracts, non-peer-reviewed preprints, studies lacking genetic confirmation, articles without English full text, and studies focused exclusively on iron-accumulation disorders outside the NBIA spectrum (e.g., Wilson disease, aceruloplasminemia) were excluded unless cited for direct mechanistic comparison.

### Reference Collection and Verification

All candidate references were cross-checked against source PDFs to verify author names, publication years, journal details, and digital object identifiers (DOIs), addressing prior concerns regarding citation-reference list mismatches. Each in-text citation was reconciled against the final reference list to eliminate orphaned or unverifiable citations.

### Data Categorization and Synthesis

Extracted data were organized thematically into sections spanning genetics and epidemiology, CoA/mitochondrial metabolism, lipid peroxidation/autophagy/ferroptosis, less common subtypes, human cellular and iPSC-derived models, iron localization/neurotransmitter metabolism/imaging biomarkers, and clinical/therapeutic developments, allowing convergent mechanistic themes across genetically distinct NBIA subtypes to be synthesized rather than presented in isolation.

### Knowledge Gaps

The synthesis specifically targeted previously under-addressed areas identified through peer review, including incomplete coverage of CoPAN and MEPAN, underrepresentation of human iPSC-derived neuronal, astrocytic, and microglial model data, insufficient discussion of the subcellular compartmentalization of accumulated iron, and an outdated therapeutic evidence base.

### Novelty and Unique Contributions

This review is distinguished by its integration of recent primary studies (2020–2026) with foundational literature, comprehensive inclusion of all major and minor NBIA subtypes within a unified pathway-based framework, and consolidated treatment of iPSC-derived cellular model data across multiple NBIA forms, features not jointly present in prior published reviews.

### Theoretical and Practical Significance

Theoretically, the review reframes iron accumulation as a downstream, amplifying consequence rather than the primary driver of NBIA pathogenesis, refining the conceptual model underlying disease classification. Practically, the consolidated synthesis of cellular models, biomarkers, and therapeutic pipelines is intended to inform future trial design and translational research prioritization for this ultra-rare disease group.

### Limitations

As a narrative rather than systematic review, formal risk-of-bias assessment and meta-analytic pooling were not performed, and study selection, while structured, retains an element of subjectivity. The rarity of NBIA disorders limits the depth of available clinical and cellular data for some subtypes (e.g., MEPAN, FAHN), and rapidly evolving iPSC-based and therapeutic literature means some very recent findings may not be captured at the time of submission.

## NBIA Landscape, Genetics and Epidemiology

Neurodegeneration with brain iron accumulation (NBIA) comprises a group of rare, progressive neurodegenerative movement disorders in which recent cohort, population-genetic, clinical, and case-based studies have begun to define subtype frequencies, natural history, and genetic architecture more quantitatively, as highlighted in **Table 1.** These studies collectively describe how specific NBIA subtypes present in different clinical settings and populations, and provide estimates of disease burden and survival that are directly relevant for counselling and for designing future disease-modifying trials.

**Table 1.** Genetics and epidemiology of NBIA disorders.

Reference	Study design / population	NBIA subtype(s)	Main findings
(Amini et al., 2024)	Retrospective cohort (122 genetically confirmed patients)	PKAN, PLAN, MPAN, BPAN, CoPAN, FAHN, KRS, aceruloplasminemia	Described clinical spectrum, MRI findings, genotype–phenotype correlations, survival, and predictors of disease progression.
(Kolarova et al., 2022)	Population genomic database analysis	13 autosomal recessive NBIA disorders	Estimated lifetime disease risk, subtype distribution, founder effects, and population-specific prevalence.
(Khan et al., 2025)	Whole-exome sequencing in one consanguineous family	REPS1-associated NBIA	Identified a novel REPS1 pathogenic variant and expanded the genetic spectrum of NBIA.
(Si et al., 2023)	Clinical, genetic and bioinformatic analysis (84 BIAD patients)	NBIA and NBIA-like disorders	Identified pathogenic variants and highlighted common pathways involving mitochondrial dysfunction, autophagy, lipid metabolism, and ferroptosis.
(Bhardwaj et al., 2021)	Retrospective pediatric cohort (27 patients)	PLAN, PKAN, MPAN, BPAN, KRS	Reported clinical characteristics, MRI features, and genetic findings in an Indian pediatric cohort.
(Sait et al., 2023)	Multicenter observational study (27 genetically confirmed patients)	PLAN, PKAN, FAHN, MPAN, CoPAN	Described clinical and molecular spectrum, identified novel variants, and showed that iron deposition may be absent during early disease stages.
(Courtois et al., 2024)	Case report with functional validation	MT-CO2-associated NBIA	Reported the first mitochondrial DNA-associated NBIA phenotype caused by an MT-CO2 variant.

In a retrospective single-center cohort study, Amini et al. analyzed 122 genetically confirmed NBIA patients from a national movement disorders referral clinic in Iran, encompassing nine subtypes (PKAN, PLAN, MPAN, BPAN, KRS, Woodhouse–Sakati syndrome, aceruloplasminemia, CoPAN, FAHN), with the main aim of estimating ambulation preservation and survival probabilities and identifying clinical predictors of loss of walking ability and death. PKAN was the most frequent subtype (57/122), followed by MPAN (23/122) and KRS (17/122), and 78.7% of patients had first-cousin consanguinity, with over half having affected siblings; almost all patients were homozygous for the causative gene except BPAN, which showed heterozygous de novo WDR45 variants, and recurrent alleles such as PANK2 c.1069C > T and ATP13A2 c.2455C > T were common in PKAN and KRS. MRI typically showed basal ganglia iron deposition with globus pallidus/substantia nigra hypointensity and an “eye-of-the-tiger” sign in PKAN, together with cerebellar and corpus callosum atrophy and white-matter changes in PLAN, more extensive iron deposition in aceruloplasminemia, and notably no brain iron accumulation in KRS, which instead displayed cerebellar/global atrophy or nonspecific white-matter lesions. Clinically, gait disorder was the most frequent presenting symptom (67.2%), progressing to gait disturbance in 80.3% and speech disorder in 45.1% over time, and spasticity, generalized dystonia, cognitive and behavioural disturbances, oculomotor abnormalities, seizures, and optic atrophy were variably distributed across subtypes; survival and ambulation analyses showed rapid progression to wheelchair use in classical PKAN and MPAN, slower progression to death in MPAN, much more protracted ambulation and survival in atypical PKAN, longest ambulation preservation and best survival in KRS, and never-ambulatory status with shortest survival in infantile PLAN, with spasticity emerging as the only independent predictor of mortality in Cox regression (Amini et al., 2024).

Using a different approach, Kolarova et al. estimated lifetime risk and carrier frequencies of 13 autosomal-recessive NBIA disorders by collecting pathogenic and likely pathogenic variants from ClinVar, HGMD, gnomAD, and an in-house exome database and calculating disease risk from allele frequencies under Hardy–Weinberg equilibrium. Across the global gnomAD dataset, the European non-Finnish dataset, and the in-house database, they found very similar combined lifetime risks of NBIA (0.88, 0.92, and 0.90 per 100,000, respectively), indicating that NBIA is ultra-rare but likely more frequent than earlier case-based estimates suggested. In this analysis, PLA2G6-associated neurodegeneration (PLAN) had the highest lifetime risk in all datasets, followed by PANK2-related PKAN and COASY-related CoPAN, together accounting for up to 75% of the combined NBIA lifetime risk, whereas recently described disorders such as GTPBP2- and REPS1-related NBIA had the lowest estimated risks. Kolarova et al. also reported population differences, including higher predicted risk of PKAN in East Asian and South Asian populations, higher ACP risk in East Asians, and an Ashkenazi Jewish founder effect for ATP13A2-related Kufor–Rakeb syndrome, and observed that more recently identified NBIA genes are under-represented in databases, with depletion of reported loss-of-function variants in genes such as COASY, CRAT, and REPS1 possibly indicating hypomorphic alleles and prenatal lethality of complete loss-of-function states (Kolarova et al., 2022). Together, (Amini et al., 2024) and (Kolarova et al., 2022) show that PKAN, MPAN, KRS, PLAN, and CoPAN are major contributors to NBIA in clinical cohorts and genetic datasets, and that consanguinity, founder variants, and ancestry-specific allele frequencies are important determinants of which subtypes predominate in different populations.

Two Indian pediatric series provide additional information on NBIA subtype distribution and clinical features in early-onset cohorts. Bhardwaj et al. retrospectively characterized 27 pediatric NBIA patients seen between 2014 and 2020, reporting PLAN as the most common subtype (13 cases), followed by PKAN (9), MPAN (2), BPAN (2), and ATP13A2-related Kufor–Rakeb syndrome (1). In this cohort, 50% of patients presented with developmental regression, one-third had epilepsy, pyramidal signs were the most frequent examination finding (89%), followed by ocular abnormalities (81%) and movement disorders (50%), and PKAN cases typically presented in the first decade with gait difficulty, frequent falls, limb dystonia, the classical “eye-of-the-tiger” MRI sign, and universal pigmentary retinopathy. PLAN cases, in contrast, showed infantile or early-childhood neuroregression with cerebellar atrophy, nystagmus or strabismus, optic atrophy, axial hypotonia, and severe motor disability; MRI was abnormal in 24/27 patients, with cerebellar atrophy as the commonest finding (52%), globus pallidus hypointensities in 44%, and in PLAN specifically, early cerebellar atrophy with widened folia, claval hypertrophy, cerebellar T2 hyperintensity, and only occasional pallidal iron at young ages. Genetic testing identified homozygous or compound-heterozygous variants in PLA2G6, PANK2, C19orf12, WDR45, and ATP13A2, confirming co-existence of several NBIA subtypes in this region and leading the authors to emphasize that NBIA should be strongly suspected in children with neuroregression and visual abnormalities, plus pyramidal or movement disorders, with MRI and ophthalmologic assessment guiding targeted genetic testing (Bhardwaj et al., 2021). In a multicenter observational study of 27 genetically confirmed NBIA patients from 20 unrelated Indian families, Sait et al. likewise found PLA2G6-associated neurodegeneration (PLAN) to be the most frequent subtype (13 individuals, mainly infantile neuroaxonal dystrophy), followed by FAHN (8 individuals), PKAN (3), CoPAN (2 neonates with prenatal-onset pontocerebellar hypoplasia), and a single juvenile-onset MPAN case. Consanguinity was reported in only 20% of families, yet 65% of patients carried homozygous variants, and more than half had recurrence in siblings, underscoring the impact of endogamy; clinically, abnormal cognition (∼81%) and extrapyramidal symptoms (∼62%) were common overall, but the detailed phenotype varied by subtype. Iron-sensitive MRI sequences (SWI/GRE) were available in 17 individuals, and basal ganglia iron deposition was identified in about one-third (6/17), particularly in older PLAN, FAHN, PKAN, and MPAN patients, whereas early-onset, severe PLAN and CoPAN cases typically lacked visible iron despite pathogenic variants in NBIA genes. Exome sequencing with CNV calling identified 22 very rare pathogenic or likely pathogenic variants across PLA2G6, FA2H, PANK2, C19orf12, and COASY, including nine novel variants and a recurrent multi-exonic PLA2G6 duplication and Sait et al. concluded that PLAN appears to be the predominant molecularly confirmed NBIA subtype in Indian pediatric cohorts and that iron deposition may be absent during early disease stages (Sait et al., 2023). Taken together, (Bhardwaj et al., 2021) and (Sait et al., 2023) complement (Amini et al., 2024) and (Kolarova et al., 2022) by showing that PLAN can be the leading NBIA subtype in pediatric series with a focus on early-onset disease, and by illustrating that cerebellar atrophy and severe neuroregression may precede or occur without early basal ganglia iron accumulation.

Additional studies expand the NBIA spectrum to include newer NBIA-related genes and NBIA-like iron disorders. In a consanguineous Pakistani family with three siblings presenting with progressive extrapyramidal symptoms, childhood-onset gait disturbance, spasticity, limb weakness, developmental delay, craniofacial features, and MRI evidence of basal ganglia iron deposition, Khan et al. used whole-exome sequencing (GRCh37/hg19) and in silico structural modelling to identify a novel homozygous missense REPS1 variant, NM_001286611.1:c.1460A > C (p.Lys487Thr), which segregated in homozygous form in all affected siblings and was absent in unaffected relatives. Multiple prediction tools (SIFT, PolyPhen-2, MutationTaster, PhD-SNP) supported a damaging effect, and the variant was classified as of uncertain significance by ACMG (American College of Medical Genetics and Genomics) criteria; conservation analysis showed that Lys487 is highly conserved across species, and homology-based 3D modelling with I-TASSER, Verify-3D, Chimera, DUET, and mCSM suggested that the Lys487Thr substitution destabilizes core structural elements and perturbs residues critical for REPS1’s role in endocytic trafficking and signalling complexes, potentially compromising neuronal endosomal–lysosomal function. Based on these data, Khan et al. argued that their family expands both the allelic and geographic spectrum of REPS1-associated NBIA (Khan et al., 2025). In a broader clinical and genetic series, Si et al. systematically characterized 84 patients with brain iron accumulation disorders (BIADs) using susceptibility-weighted MRI, extensive genetic testing (whole-exome sequencing, repeat-expansion assays, mtDNA sequencing, CNV analysis), and pathway-level bioinformatics to define the mutational spectrum and shared mechanisms underlying NBIA and NBIA-like phenotypes. They found that 30 patients (35.7%) carried pathogenic or likely pathogenic variants, including six with classic NBIA gene pathogenic variants (PANK2, PLA2G6, FTL) causing PKAN, PLAN, and neuroferritinopathy, while others had variants in NBIA-like genes (e.g. SCP2, AP4M1, GTPBP2, REPS1, CRAT), neurogenetic disease genes (ABCD1, ARSA, COQ2, GBA, PRKN, FBXO7, VPS13A, ATN1, ATXN2/3, CACNA1A, HTT), or novel loci. Iron deposition most frequently involved the globus pallidus, substantia nigra, and dentate nucleus, with additional involvement of putamen, red nucleus, and caudate, and widespread cortical and cerebellar atrophy were common in both genetic and non-genetic cases; family history was strongly associated with mutation detection, and age at onset tended to be younger in patients with non-dynamic (point/indel) pathogenic variants than in those with repeat expansions or no identified mutation. Gene Ontology and KEGG enrichment analyses of 35 BIAD-associated genes (10 confirmed NBIA, 7 NBIA-like, and additional non-NBIA genes plus FXN) indicated clustering in autophagy, mitochondrial organization, lipid biosynthesis and metabolism, lysosomal and vesicular compartments, transition-metal ion homeostasis, and ferroptosis-related pathways. Si et al. concluded that NBIA genes and other BIAD-associated genes participate in overlapping biological processes rather than forming isolated disease pathways, and provided a systems-level framework for BIADs that includes NBIA disorders (Si et al., 2023).

Finally, Courtois et al. reported a 34-year-old woman with a progressive mitochondrial disease presenting with an NBIA-like phenotype, in whom they identified a novel heteroplasmic MT-CO2 variant (m.8091G > A, p.Gly169Asp) that causes complex IV deficiency and intracerebral iron accumulation. The patient had normal early development followed by childhood-onset myopia and bilateral sensorineural hearing loss, adolescent-onset static and kinetic ataxia with tetrapyramidal signs, and progressive cognitive decline in her twenties; MRI at age 32 showed marked cortico-subcortical and infratentorial (vermal) atrophy with pronounced T2 hypointensity in the basal ganglia and central calcifications, and optic atrophy on fundoscopy, consistent with an NBIA-like neuroradiological pattern. Extensive nuclear genetic testing, including a hereditary spastic paraplegia panel, nine NBIA genes, PEO1, POLG, and whole-genome sequencing, was negative, but mitochondrial DNA sequencing from muscle revealed the previously undescribed MT-CO2 variant at 96% heteroplasmy (and ∼10–12% in fibroblasts and urine), absent from population databases and affecting a highly conserved residue. In silico structural analysis with DynaMut2 showed that Gly169Asp introduces multiple new inter-subunit interactions and destabilizes COX2 (predicted ΔΔG −0.59 kcal/mol), and functional studies demonstrated severely reduced complex IV specific activity in muscle with preserved citrate synthase, decreased COX2 protein levels in fibroblasts, and globally impaired mitochondrial respiration (reduced basal, ATP-linked, leak, and maximal oxygen consumption rates), confirming a pathogenic complex IV defect. Courtois et al. noted that prior MT-CO2 pathogenic variants had been linked to ataxia, neuropathy, and cerebellar changes without brain iron deposition and proposed that this variant establishes MT-CO2-related, mitochondrially encoded NBIA within the broader NBIA spectrum, underscoring the importance of including mtDNA sequencing when nuclear NBIA panels are unrevealing (Courtois et al., 2024).

Overall, these studies provide complementary perspectives on NBIA genetics and epidemiology: (Amini et al., 2024) offer detailed natural-history and survival data from a high-consanguinity clinical cohort; (Kolarova et al., 2022) supply lifetime-risk estimates and carrier frequencies for autosomal-recessive NBIA disorders from large genetic databases;, (Bhardwaj et al., 2021) and (Sait et al., 2023) describe subtype distribution, clinical features, and MRI patterns in Indian pediatric cohorts with an emphasis on PLAN and early-onset disease; (Khan et al., 2025) expand the NBIA spectrum to include REPS1-associated disease in a consanguineous family; (Si et al., 2023) situate NBIA within a wider BIAD framework with overlapping genetic and pathway-level findings; and (Courtois et al., 2024) introduce MT-CO2-associated, mitochondrially encoded NBIA. Limitations across these studies include modest sample sizes for rarer NBIA subtypes, reliance on tertiary referral cohorts, incomplete variant reporting in some series, and the evolving nature of NBIA gene discovery, which means that estimates for newer genes may change as more data become available. From a translational perspective, these data collectively refine the current understanding of NBIA subtype frequencies, natural history, imaging signatures, and genetic architecture and highlight the need for larger international cohorts, systematic inclusion of mtDNA sequencing in NBIA-like presentations, and continued integration of clinical, imaging, and genetic information to support future mechanistic and therapeutic work.

## CoA and Mitochondrial Metabolism (PKAN, CoPAN, MEPAN)

Coenzyme A (CoA) biosynthesis and mitochondrial metabolism represent a central pathogenic axis across several NBIA subtypes, particularly pantothenate kinase–associated neurodegeneration (PKAN), COASY protein–associated neurodegeneration (CoPAN), and mitochondrial enoyl-CoA reductase protein–associated neurodegeneration (MEPAN), where defects in PANK2, COASY, and MECR converge on mitochondrial dysfunction, disturbed iron handling, and progressive motor disease, as highlighted in **Table 2**. Recent cellular, animal, and clinical studies collectively describe how CoA and mitochondrial pathways are disrupted in these disorders and provide initial evidence for candidate therapeutic strategies, while also illustrating that some models show early mitochondrial and functional impairment before overt iron accumulation or neurodegeneration.

**Table 2.** CoA Biosynthesis and mitochondrial dysfunction in PKAN, CoPAN, and MEPAN.

Reference	Disease / gene	Experimental model	Main findings
(Di Meo et al., 2020)	CoPAN (COASY)	Neuron-specific Coasy knockout mouse	Demonstrated early mitochondrial dysfunction, iron dyshomeostasis, and motor deficits preceding overt neurodegeneration.
(Álvarez-Córdoba et al., 2021)	PKAN (PANK2)	Patient fibroblasts and induced neurons	Showed impaired mitochondrial phosphopantetheinylation, Fe–S cluster biogenesis, respiratory complex I function, and partial rescue with pantothenate.
(Álvarez-Córdoba et al., 2022)	PKAN (PANK2)	Patient-derived fibroblasts	Nutritional supplements improved mitochondrial function, reduced iron accumulation and oxidative stress in cells with residual PANK2 activity.
(Nataraj et al., 2023)	MEPAN (MECR)	Two genetically confirmed patients	Individualized deep-brain stimulation improved refractory dystonia and motor function.
(Cozzi et al., 2025)	CoPAN (COASY)	Patient fibroblasts, hiPSC-derived astrocytes	Identified mitochondrial dysfunction, iron accumulation, cellular senescence and ferroptosis; CoA and leriglitazone improved cellular defects.
(Murdock et al., 2025)	MEPAN (MECR)	CRISPR knock-in mouse model	Demonstrated defective mitochondrial fatty acid synthesis, impaired Fe–S cluster biogenesis, respiratory supercomplex instability and neurological dysfunction.
(Shao et al., 2025)	CoPAN (COASY)	*Drosophila* model	Showed CoA deficiency causes mitochondrial dysfunction, ATP depletion and neurodegeneration; human COASY rescued disease phenotypes.
(Toczylowska et al., 2025)	PKAN (PANK2)	Serum metabolomic/ metallomic analysis	Identified metabolic alterations associated with mitochondrial dysfunction, oxidative stress and potential ferroptosis.
(Cavestro et al., 2026)	CoPAN (COASY)	Inducible neuron-specific knockout mouse	Demonstrated neuroinflammation as a major pathogenic mechanism and therapeutic benefit of leriglitazone.

In CoPAN, Cozzi et al. used patient-derived fibroblasts and hiPSC-derived astrocytes from individuals carrying compound-heterozygous p.Arg499Cys + p.Gln59 or homozygous p.Arg499Cys COASY variants to dissect iron dyshomeostasis, mitochondrial dysfunction, vesicular trafficking, senescence, and ferroptosis. They showed that CoPAN fibroblasts have Perls-positive iron aggregates, increased cytosolic labile iron, elevated H-ferritin, reduced transferrin receptor 1 (TfR1), unchanged ferroportin and DMT1-IRE, reduced viability, and markedly decreased α-acetylated tubulin that depends on iron and can be partially restored by CoA and more effectively by leriglitazone, with global lysine acetylation preserved. In hiPSC-derived astrocytes, Cozzi et al. observed swollen mitochondria with damaged cristae, mutation-dependent alterations in SynaptoZip/Synbond vesicular trafficking, much higher iron accumulation than in fibroblasts (∼80% and 35% Perls-positive cells vs ∼3% in controls), reduction of iron-positive cells with CoA supplementation, decreased TfR1, a pronounced senescent phenotype in CoPAN[Arg499Cys + Gln59*] astrocytes (∼80% SA-β-gal-positive vs ∼2% in CoPAN[Arg499Cys] and controls), and a strong correlation between Perls-positive and SA-β-gal-positive cells linking iron accumulation to senescence. Western blot analyses showed decreased GPX4 and increased malondialdehyde (MDA) only in CoPAN astrocytes, with reduced NCOA4 in CoPAN cells independent of cell type and no disease-specific changes in 4HNE, indicating that astrocytes, but not fibroblasts, more clearly exhibit lipid peroxidation and ferroptotic features; Cozzi et al. proposed that CoA impairment and mitochondrial dysfunction drive cytosolic iron accumulation in astrocytes, leading to senescence and susceptibility to ferroptosis, and suggested that astrocytes may more faithfully recapitulate CoPAN pathology than fibroblasts and that therapeutic strategies should focus on improving mitochondrial health and CoA supply rather than COASY expression via IRE/IRP (Cozzi et al., 2025).

Complementing these patient-cell data, Di Meo et al. developed a conditional neuron-specific Coasy knockout (Syn-Coasy) mouse using Synapsin-1 Cre-loxP, showing growth retardation from postnatal day 8, progressive sensorimotor impairment, dystonia-like movements, abnormal gait, loss of postural control, limb rigidity, tail stiffness, and premature death around postnatal day 13, but no neuronal loss, neuroinflammation, or intracellular inclusions, suggesting functional neuronal impairment precedes overt neurodegeneration. Biochemical and imaging studies demonstrated unchanged brain CoA, dpCoA, acetyl-CoA, and global protein acetylation with pantothenate accumulation, increased FtL, reduced TfR1, decreased DMT1, minimal Perls-detectable iron, diffuse elevations of iron, calcium, and magnesium by TOF-SIMS, selective ferritin accumulation in cortical and subcortical GABAergic interneurons, impaired pyruvate-dependent mitochondrial respiration, abnormal mitochondrial ultrastructure, increased autophagosomes and mitophagosomes, and altered membrane organization, while individual respiratory chain complex activities remained largely preserved and pyruvate dehydrogenase activity was modestly increased (Di Meo et al., 2020). At an invertebrate level, Shao et al. generated Drosophila melanogaster models with tissue-specific knockdown of CoASY (Dpck), showing reduced CoA levels, severe developmental abnormalities, impaired climbing, muscle degeneration, and, with neuronal knockdown, reduced CoA and ATP, decreased mitochondrial membrane potential, locomotor deficits, brain vacuolization, neuronal apoptosis, and dopaminergic PPL1 neuron loss. Mechanistic analyses identified mitochondrial aggregation, reduced ATP5a, impaired electron transport chain complexes I and III, disrupted ETC assembly, decreased ETC subunit expression, marked ATP depletion, mtDNA release, elevated extra-mitochondrial TFAM, and apoptosis, all rescued by transgenic human COASY, while iron dysregulation was not observed, and Shao et al. concluded that CoA deficiency primarily impairs mitochondrial integrity and energy production, with brain iron accumulation in human CoPAN likely a secondary event (Shao et al., 2025).

To further explore CoPAN pathobiology and therapy, Cavestro et al. developed an inducible neuron-specific Coasy knockout mouse (SLICK-Coasy) and evaluated the brain-penetrant PPARγ agonist leriglitazone. Unlike constitutive models, SLICK-Coasy mice reproduced key clinical features of CoPAN, including progressive motor impairment, reduced body weight, decreased survival, neurodegeneration, neuroinflammation, and brain iron dyshomeostasis, with neuronal Coasy ablation causing increased ferritin light chain (FtL) and altered iron homeostasis. Histopathology showed neuronal loss, spongiform vacuolation, eosinophilic neurons, increased plasma neurofilament light (NfL), astrocytosis, microglial activation, and elevated TNFα, IL1β, and IL6, while RNA-seq revealed hundreds of differentially expressed genes enriched in immune and inflammatory pathways, cytokine–receptor interactions, apoptosis, and impaired neuronal development. Leriglitazone, administered after neuronal Coasy deletion, significantly improved motor performance, reduced ferritin accumulation, decreased plasma NfL, attenuated astrocyte and microglial activation, lowered pro-inflammatory cytokine expression and neuroinflammation-associated genes, and restored iron homeostasis, though it did not improve survival or restore global brain CoA levels; Cavestro et al. concluded that neuroinflammation is a major pathogenic component of CoPAN and that PPARγ activation can counteract neurodegeneration and neuroinflammation (Cavestro et al., 2026). Taken together, (Cozzi et al., 2025), (Di Meo et al., 2020), (Shao et al., 2025) and (Cavestro et al., 2026) consistently link COASY deficiency to CoA-related mitochondrial dysfunction, iron dyshomeostasis, and motor impairment across human cells, mouse, and fly models and provide initial support for CoA supplementation and PPARγ-directed therapies.

In PKAN, Álvarez-Córdoba et al. examined how PANK2 pathogenic variants disrupt mitochondrial metabolism by impairing mitochondrial phosphopantetheinyl-proteins in patient fibroblasts and induced neurons, focusing on mtACP, AASS, ALDH1L2, protein lipoylation of PDH and KGDH, Fe–S cluster biogenesis (NFS1, ISD11, LYRM4), aconitase activities, and respiratory complex I assembly (MT-ND1, NDUFA9). They found marked reductions in mtACP, AASS, and ALDH1L2 with preserved cytosolic phosphopantetheinyl-proteins, reduced lipoylation of PDH and KGDH, decreased PDH activity, downregulation of Fe–S cluster components, reduced cytosolic and mitochondrial aconitase, and defective complex I assembly and activity; treatment with 500 μM pantothenate restored PANK2 expression and transcript levels, normalized mitochondrial phosphopantetheinyl-proteins, improved lipoylation, partially rescued PDH, aconitase, and complex I activities and Fe–S cluster protein expression in responder fibroblasts retaining residual PANK2 protein, and reduced Prussian Blue-positive iron while restoring PANK2 and mtACP in induced neurons (Álvarez-Córdoba et al., 2021). Building on this, Álvarez-Córdoba et al. tested pantothenate, pantethine, vitamin E, omega-3 fatty acids, L-carnitine, and thiamine in PKAN fibroblasts, showing that all six supplements reduced intracellular iron, restored morphology, increased PANK2, mtACP, and NFS1 expression, improved mitochondrial phosphopantetheinylation and Fe–S cluster biogenesis, enhanced PANK2 transcription via NF-Y, FOXN4, and hnRNPA/B, restored PGC-1α, phosphorylated PGC-1α, and TFAM, reduced lipid peroxidation, and partially rescued PDH and complex I, with effects restricted to cells with residual PANK2 (Álvarez-Córdoba et al., 2022). In parallel, Toczylowska et al. analyzed serum metabolomic and metallomic profiles in 12 adult PKAN patients and 12 matched controls by ^1H NMR and mass spectrometry, reporting a distinct PKAN profile with a 100–500-fold increase in serum citrate, alterations in metabolites linked to energy, amino acid and lipid metabolism, neurotransmission, and oxidative stress, changes in lipid-related molecules (increased phosphatidylethanolamine, reduced fatty acids, PUFAs, MUFAs, cholesterol, cholesterol esters), and multiple metals (decreased iron, zinc, selenium, magnesium, potassium, strontium; increased cobalt, manganese, boron), together with three metabolic subgroups and significant correlations between PKAN Disability Rating Scale scores and selected metabolites and metals. Toczylowska et al. concluded that PKAN is associated with oxidative stress, impaired energy production, altered neurotransmission, and membrane dysfunction and proposed elevated citrate and metal disturbances as potential contributors to ferroptosis, with this mechanism needing further experimental validation. (Toczylowska et al., 2025) In addition, Álvarez-Córdoba et al. suggested that commercially available supplements may represent safe therapeutic candidates for PKAN patients with residual PANK2 activity, while noting that truncating pathogenic variants with complete loss of PANK2 showed no response (Álvarez-Córdoba et al., 2022). Together, these PKAN studies indicate that PANK2 deficiency selectively impairs mitochondrial phosphopantetheinylation, lipoic acid–dependent enzymes, Fe–S cluster biogenesis, and complex I, and that pantothenate-based and multimodal supplementation can partially correct these defects in mutation-specific contexts.

In MEPAN, Murdock et al. developed compound-heterozygous Mecr mutant mice carrying Y285C and truncating alleles to model MECR-related mitochondrial fatty acid synthesis (mtFASII) defects, and showed early-onset movement abnormalities, impaired coordination, ataxic gait, muscle weakness, balance deficits, optic neuropathy, olfactory dysfunction, reduced body weight, and defective motor performance, with preserved gross brain morphology and Purkinje cell numbers, indicating neuronal dysfunction without overt neurodegeneration. Biochemical analyses demonstrated reduced MECR protein, markedly impaired protein lipoylation, selective cerebellar mitochondrial oxidative phosphorylation deficiency with reduced complex I/II-linked respiration and maximal capacity, reductions in ISC assembly proteins (LYRM4, NFS1, ISCU) and multiple complex I subunits, defective acylation of mitochondrial ACP and disrupted ACP–LYRM interactions, defective assembly of OXPHOS complexes and respiratory supercomplexes (complexes I, III, IV), reduced supercomplex stability, altered ATP synthase organization, decreased succinate dehydrogenase activity, ∼50% reduction in mitochondrial aconitase, and defective lipoylation of pyruvate dehydrogenase and glycine cleavage system proteins. Histological and proteomic studies showed region-specific MECR expression in the brain and retina, with retinal pathology but relatively preserved retinal protein lipoylation, leading Murdock et al. to conclude that mtFASII maintains mitochondrial bioenergetics via acylated ACP for ISC machinery and respiratory supercomplex assembly and that MECR loss destabilizes both ISC biogenesis and OXPHOS supercomplexes, causing defective ATP production and progressive neurological dysfunction (Murdock et al., 2025). Clinically, Nataraj et al. retrospectively evaluated deep brain stimulation (DBS) in two pediatric siblings with genetically confirmed MEPAN and severe generalized dystonia refractory to multiple pharmacologic therapies, using temporary stereo electroencephalography depth electrodes in GPi, VIM, Vo, VA, STN, and PPN to identify patient-specific targets before permanent implantation. One patient received GPi and PPN stimulation, the other GPi and VIM, and at approximately 12 months postoperatively both showed substantial motor improvement, with 34.9% and 49.6% reductions in Burke–Fahn–Marsden dystonia motor scores, a 19% improvement in Barry–Albright scores in one patient, and subjective gains in posture, mobility, feeding, communication, and quality of life, without perioperative or postoperative complications; Nataraj et al. **c**oncluded that DBS is a promising symptomatic treatment for refractory dystonia in MEPAN and emphasized individualized target selection (Nataraj et al., 2023).

Overall, the available data across PKAN, CoPAN, and MEPAN indicate that genetically defined defects in CoA biosynthesis and related mitochondrial pathways frequently coexist with iron‑handling abnormalities, disturbed energy metabolism, and motor or dystonia phenotypes in cellular and animal models as well as in small clinical series. Several of these studies also report beneficial effects of experimental interventions, such as CoA and nutrient supplementation, PPARγ activation with leriglitazone, and deep brain stimulation, on selected cellular, biochemical, or clinical readouts, although these findings remain preliminary and are based on limited sample sizes. Important constraints include small cohorts in clinical and supplementation studies, model‑specific differences in iron accumulation and oxidative stress, partial correction of biochemical defects without recovery of survival or global CoA levels in some interventions, and the absence of controlled trials to establish efficacy and safety for approaches such as leriglitazone, nutritional supplementation, and DBS. Taken together, these studies justify further work on CoA biosynthesis, mitochondrial phosphopantetheinylation, mtFASII, and related pathways as candidate mechanistic targets in NBIA subtypes, and underscore the need to integrate cellular, animal, and clinical evidence when designing future mechanistic experiments and early‑phase therapeutic studies.

## Lipid Peroxidation, Autophagy and Ferroptosis

Lipid peroxidation, impaired autophagy–lysosomal function, and ferroptotic susceptibility have emerged as key cellular themes in β-propeller protein-associated neurodegeneration (BPAN), a WDR45-related NBIA subtype, across multiple patient-derived fibroblast studies and detailed genetic analyses, as highlighted in **Table 3.** Recent studies show that WDR45 loss or dysfunction consistently affects autophagy–lysosome pathways, iron handling, and lipid oxidation, while providing early evidence that lysosomal-targeted interventions such as L-serine may offer more comprehensive correction of BPAN-associated cellular pathology than iron chelation alone.

**Table 3.** Lipid peroxidation, autophagy, and ferroptosis in BPAN.

Reference	Experimental model	Major pathway investigated	Main findings
(Diaw et al., 2022)	Patient-derived fibroblasts	Autophagy, ferritinophagy, ferroptosis	WDR45 deficiency impaired autophagy, ferritinophagy, lysosomal function, and increased susceptibility to ferroptosis.
(Suárez-Carrillo et al., 2023)	Patient-derived fibroblasts	Autophagy, mitochondrial dysfunction, lipid peroxidation	Demonstrated defective autophagy, mitochondrial dysfunction, iron accumulation, and increased lipid peroxidation; antioxidants reduced oxidative damage but did not restore autophagy.
(H. E. Lee et al., 2024)	Patient-derived fibroblasts	Lysosomal dysfunction and lipid peroxidation	L-serine restored lysosomal function, reduced iron accumulation, lipofuscin formation, and oxidative stress more effectively than iron chelation.
(Peng et al., 2024)	Clinical case and molecular analyses	WDR45 mutation and iron homeostasis	Identified a novel WDR45 mutation causing nonsense-associated splicing alteration (NASA), impaired autophagy, and dysregulated iron transport pathways.

In patient fibroblasts, Suárez-Carrillo et al. examined WDR45 pathogenic variants in two BPAN cases to characterize defects in autophagy, iron homeostasis, lipid peroxidation, mitochondrial function, and responses to selected antioxidants. They showed marked reductions in WDR45 protein and mRNA, decreased expression of multiple autophagy-related proteins (ULK1, BECN1, ATG12–ATG5, LC3B, p62, TFEB), reduced lysosomal proteins (LAMP1, LAMP2, GBA1, CATC), impaired autophagosome formation, defective autophagic flux, and generalized autophagy–lysosomal dysfunction. BPAN fibroblasts had significantly increased intracellular iron by Prussian Blue and ICP-MS, which was markedly reduced by recombinant WDR45 expression, supporting a direct link between WDR45 deficiency, autophagy impairment, and iron dysregulation. Mutant cells accumulated lipofuscin-like aggregates shown by autofluorescence, Sudan Black, spectral confocal microscopy, and TEM, together with mitochondrial vacuolization, damaged mitochondrial membranes, and dilated rough ER, indicating defective degradation and cellular stress. Expression of proteins regulating iron trafficking, storage, and Fe–S metabolism (SLC40A1, TFR1, DMT1, ferritin, MFRN2, FTMT, NCOA4, FXN, NFS1, ISCU, IRP1) was reduced, with increased labile iron, demonstrating widespread iron-metabolism disruption; mitochondrial bioenergetics were impaired, with reduced basal and maximal respiration, diminished ATP, decreased spare respiratory capacity, fragmented networks, reduced respiratory proteins, and increased mitochondrial lipid peroxidation. Pantothenate, vitamin E, and α-lipoic acid significantly prevented iron accumulation and lipid peroxidation but did not restore WDR45 expression, correct autophagy defects, or improve bioenergetics, indicating only partial protection (Suárez-Carrillo et al., 2023).

In a complementary patient-fibroblast study, Diaw et al. analyzed a newly identified BPAN patient with a missense WDR45 variant p.Asn61Lys, generalized dystonia, anarthria, Parkinsonism, spasticity, stereotypies, developmental delay, and MRI evidence of brain atrophy with iron deposition in globus pallidus and substantia nigra. Mutant fibroblasts showed reduced WDR45 mRNA, impaired lysosomal integrity (decreased LAMP1/2, GBA, GAA), reduced TOMM20 and fragmented mitochondrial networks, and defective autophagy with reduced autophagosome formation and impaired LC3-II accumulation after bafilomycin A1, while Beclin1 remained unchanged, suggesting a defect at intermediate WDR45-dependent autophagosome maturation rather than initiation. Diaw et al. found decreased basal GPX4, FTH, and p62, abnormal responses to the ferroptosis inducer RSL3, and disrupted ferritinophagy: control fibroblasts showed dose-dependent NCOA4 increases after ferric ammonium citrate, whereas WDR45-mutant cells did not, indicating impaired ferritin-mediated iron recycling; they concluded that WDR45 loss-of-function causes defective autophagy that disrupts ferritinophagy, mitophagy, lysosomal and mitochondrial integrity, iron recycling, and ferroptosis, providing a mechanistic explanation for abnormal iron accumulation and neurodegeneration in BPAN (Diaw et al., 2022).

Therapeutic modulation of lysosomal function was examined by H. E. Lee et al., who tested L-serine in WDR45-mutant BPAN fibroblasts to see whether lysosomal dysfunction, iron accumulation, and oxidative stress could be ameliorated. Using confocal microscopy, TEM, cryo-ET, CLEM, EDX, lysosomal activity assays, RT-qPCR, and ROS measurements, they showed that enlarged lysosomes in BPAN cells had reduced lysosomal enzyme activity and accumulated ferrous iron, oxidized lipids, cholesterol, and lipofuscin, indicating severe lysosomal dysfunction and oxidative damage. The iron chelator 2,2′-bipyridine partially reduced cytoplasmic iron but did not eliminate lysosomal iron, restore lysosomal function, normalize oxidative-stress gene expression, or improve mitochondrial ROS, suggesting that iron chelation alone is insufficient. Conversely, L-serine treatment increased TFEB, CTSD, and MCOLN1 expression, reduced enlarged lysosomal vacuoles, decreased lysosomal iron and lipid accumulation, suppressed cholesterol and lipid radicals, and significantly improved lysosomal degradative activity. Cryo-ET revealed multilamellar “onion-like” lipid structures in iron-loaded lysosomes, EDX showed colocalized iron and oxygen in these aggregates, and CLEM confirmed these structures as lipofuscin; L-serine substantially reduced lipofuscin, indicating improved clearance of oxidized lipid aggregates and restoration of lysosomal homeostasis. Lee et al. concluded that lysosomal dysfunction rather than iron accumulation alone is a central driver of oxidative damage in BPAN and proposed L-serine as a promising disease-modifying candidate that restores lysosomal activity more effectively than iron chelation (H. E. Lee et al., 2024).

In a genetic and transcript-level study, Peng et al. characterized a de novo WDR45 nonsense mutation c.726C > G (p.Tyr242Ter) in a young female and examined pathogenetic mechanisms beyond classical nonsense-mediated mRNA decay. Using WES, Sanger sequencing, array-CGH, RNA-seq, mini-gene assays, western blot, and pathway/GO analyses, they identified the mutation in exon 9 causing premature truncation and reduced WDR45 expression via NMD, and, because it lies three nucleotides from the canonical splice donor site, demonstrated nonsense-associated splicing alteration (NASA) generating an abnormal transcript lacking exon 9, representing the first NASA described for WDR45. RNA-seq revealed alternative splicing and reduced WDR45 transcripts, and functional analyses showed decreased WDR45 and p62, indicating impaired autophagy, while transcriptome data revealed downregulation of iron transport genes TFRC, TFR2, SCARA5 and GO enrichment in cellular iron homeostasis, iron transport, ferric/ferrous binding, and transferrin receptor activity. Pathway analyses also identified immune-related changes and altered expression of several NBIA-associated genes (PANK2, PLA2G6, COASY, C19orf12, REPS1); clinically, the patient had developmental delay, intellectual disability, language impairment, and febrile seizures without detectable brain iron on MRI, highlighting that WDR45 pathogenic variants can be diagnosed genetically before classical imaging features appear (Peng et al., 2024).

Taken together (Suárez-Carrillo et al., 2023), (Diaw et al., 2022), (H. E. Lee et al., 2024), and (Peng et al., 2024) show that WDR45 deficiency in BPAN is repeatedly associated with autophagy–lysosomal dysfunction, including reduced expression of core autophagy proteins, impaired autophagosome formation, defective autophagic flux, and decreased lysosomal membrane and enzyme markers, together with altered ferritinophagy and ferroptosis‑related proteins, such as changes in GPX4, ferritin heavy chain, p62, and NCOA4, and abnormal responses to ferroptosis induction. Across these studies, patient‑derived fibroblasts and transcriptomic data also document disrupted iron transport and storage, with increased intracellular iron or lysosomal iron retention, reduced ferritin and transferrin receptor expression, downregulation of iron transport genes including TFRC, TFR2, and SCARA5, and a higher labile iron pool, alongside lysosomal accumulation of oxidized lipids, cholesterol, and lipofuscin, and mitochondrial bioenergetic defects such as reduced respiration, ATP production, and fragmented networks. In this context, (H. E. Lee et al., 2024) identify L‑serine as a candidate lysosomal‑targeted therapy that, in BPAN fibroblasts, increases TFEB, CTSD, and MCOLN1 expression, improves lysosomal enzyme activity, reduces lysosomal iron and lipid radicals, and decreases lipofuscin accumulation more effectively than iron chelation with 2,2′‑bipyridine, while (Peng et al., 2024) demonstrate that some WDR45 nonsense pathogenic variants can produce complex transcript‑level effects, including nonsense‑mediated mRNA decay and nonsense‑associated splicing alteration (NASA), with associated downregulation of iron‑homeostasis and autophagy‑related genes, in patients who have clinical BPAN features but no visible brain iron on MRI. Important limitations of this BPAN literature include the exclusive use of fibroblast models from a small number of patients, the absence of long‑term *in vivo* validation of L‑serine or antioxidant treatments, and a lack of data from neuronal and glial cells, which may show different degrees or patterns of vulnerability. Within these constraints, the studies provide a cellular framework in which lipid peroxidation, autophagy–lysosomal impairment, ferritinophagy disruption, and ferroptotic susceptibility are closely linked to BPAN pathology and justify further work in human iPSC‑derived neurons and astrocytes, as well as early‑phase clinical investigations of therapies designed to restore lysosomal function and iron–lipid homeostasis.

## Subtypes Beyond PKAN/PLAN: MPAN, Neuroferritinopathy and FAHN

Subtypes beyond PKAN and PLAN, mitochondrial membrane protein-associated neurodegeneration (MPAN), fatty acid hydroxylase–associated neurodegeneration (FAHN), and neuroferritinopathy, illustrate how NBIA extends into disorders where mitochondrial bioenergetics, myelin integrity, and glial iron handling are disturbed in distinct but mechanistically informative ways, as highlighted in **Table 4.** Recent fibroblast, hiPSC-based, and clinical studies provide disease-relevant cellular models and describe how pathogenic variants in C19orf12, FA2H, FTL1, and FTH1 contribute to NBIA-like phenotypes.

**Table 4.** Experimental models of MPAN, neuroferritinopathy, and FAHN.

Reference	Disease / gene	Experimental model	Main findings
(Angelini et al., 2023)	MPAN (C19orf12)	Clinical, neuropathological, and fibroblast studies	Identified autosomal dominant MPAN, expanded the clinical spectrum, and demonstrated altered iron homeostasis, mitochondrial dysfunction, and impaired autophagy.
(Krogulec et al., 2024)	MPAN (C19orf12)	Patient-derived hiPSC lines	Established well-characterized hiPSC lines as a disease model for investigating MPAN mechanisms and therapeutic strategies.
(Wydrych et al., 2025)	MPAN (C19orf12)	Patient-derived fibroblasts	Demonstrated impaired mitochondrial bioenergetics, altered lipid metabolism, redox imbalance, and partial autophagy defects associated with disease severity.
(Efendic et al., 2022)	FAHN (FA2H)	Patient-derived hiPSC line	Generated the first FAHN-specific hiPSC model for studying disease mechanisms.
(Efendic et al., 2025)	FAHN (FA2H)	hiPSC-derived neuron–oligodendrocyte co-culture	Demonstrated defective myelination, reduced FA2H expression, and impaired autophagic flux.
(Moro et al., 2025)	Neuroferritinopathy (FTL1)	hiPSC-derived astrocytes	Showed that iron overload induces astrocyte reactivity, senescence, and ferroptosis through cytosolic iron accumulation.
(Rohani et al., 2026)	Neuroferritinopathy (FTH1)	Clinical and genetic study	Identified a novel FTH1 variant and expanded the genotypic and phenotypic spectrum of FTH1-related neuroferritinopathy.

Wydrych et al. investigated the metabolic consequences of pathogenic C19orf12 pathogenic variants in mitochondrial membrane protein–associated neurodegeneration (MPAN) by analyzing primary fibroblasts from 11 patients and comparing them with healthy controls, under both glucose‑ and galactose‑based culture conditions. They comprehensively assessed mitochondrial function, bioenergetics, oxidative stress, lipid metabolism, autophagy, proteomics, and metabolic adaptation. MPAN fibroblasts had significantly shorter mitochondria, reduced metabolic activity, and decreased basal, ATP‑linked, maximal, and proton leak–associated oxygen consumption, along with reduced abundance of OXPHOS complex III and ATP synthase and diminished oxidation of tricarboxylic acid (TCA) cycle substrates and fatty acids, indicating impaired mitochondrial bioenergetics without changes in mitochondrial mass. When cells were switched to an oxidative phosphorylation (OXPHOS)–promoting galactose medium, MPAN fibroblasts showed reduced metabolic flexibility, with lower proliferation, decreased metabolic activity, impaired adaptation to oxidative metabolism, and weaker increases in respiratory capacity than controls; several of these defects quantitatively correlated with clinical disease severity. The authors also found disturbed redox homeostasis, with elevated intracellular reactive oxygen species (ROS), altered antioxidant protein expression, and evidence for increased superoxide generation via a complex I/III‑dependent mechanism, but no detectable oxidative damage to proteins or lipids under their conditions, suggesting that antioxidant remodeling may be an adaptive response rather than overt oxidative stress. Lipidomic and proteomic analyses revealed altered neutral lipid and phospholipid profiles and changes in metabolic and antioxidant pathways, and autophagy studies showed that autophagic flux was partially impaired, particularly under OXPHOS‑promoting conditions, rather than completely blocked (Wydrych et al., 2025). Overall, Wydrych et al. concluded that pathogenic C19orf12 pathogenic variants cause broad disturbances in mitochondrial bioenergetics, metabolic adaptation, redox regulation, lipid metabolism, and autophagy, which become more pronounced when cells rely on OXPHOS and correlate with disease severity.

To complement the recessive MPAN fibroblast work, Angelini et al. investigated autosomal dominant mitochondrial membrane protein–associated neurodegeneration (AD‑MPAN) caused by heterozygous C19orf12 variants, aiming to expand the phenotypic spectrum and compare cellular abnormalities with autosomal recessive MPAN (AR‑MPAN). They studied eight affected individuals from four unrelated AD‑MPAN families, performing detailed clinical assessment, brain MRI, molecular genetic analysis, neuropathology in one patient, and functional studies in primary fibroblasts from AD‑MPAN, AR‑MPAN, and controls. Angelini et al. identified four truncating heterozygous C19orf12 variants, including two novel nonsense variants (p.Glu84* and p.Glu91*) in the last exon, and showed that two affected parents carried the familial variants in a somatic mosaic state, providing strong evidence for autosomal dominant inheritance. Clinically, most patients had the typical MPAN phenotype with childhood‑onset walking difficulties, dystonia, Parkinsonism, pyramidal signs, cognitive decline, optic abnormalities, and iron accumulation in the globus pallidus and substantia nigra on MRI, whereas the two mosaic individuals developed late‑onset disease (∼35 years) with rapidly progressive deterioration, one initially misdiagnosed as frontotemporal dementia with motor neuron involvement, illustrating that mosaic AD‑MPAN can mimic other neurodegenerative conditions. Neuropathological examination revealed neuronal loss, axonal spheroids, Lewy bodies, α‑synuclein‑positive inclusions, hyperphosphorylated tau‑positive neurons, and limited iron deposition mainly in the globus pallidus, consistent with previously reported MPAN pathology. In functional assays, AD‑MPAN fibroblasts showed markedly reduced C19orf12 expression, greater intracellular iron accumulation, higher ferritin heavy‑chain levels, increased mitochondrial marker VDAC1, and reduced LC3B‑II/LC3B‑I ratios, indicating more pronounced abnormalities in iron storage, mitochondrial homeostasis, and autophagy than in AR‑MPAN fibroblasts (Angelini et al., 2023). Taken together, (Angelini et al., 2023) and (Wydrych et al., 2025) show that, in addition to the broad mitochondrial and metabolic defects described in recessive MPAN, dominant and mosaic C19orf12 variants broaden the clinical spectrum to atypical adult‑onset disease and intensify iron‑storage and autophagy disturbances at the cellular level.

To provide a human stem cell platform for mechanistic studies of mitochondrial membrane protein-associated neurodegeneration (MPAN), Krogulec et al. generated patient-derived hiPSC lines from individuals carrying the homozygous C19orf12 c.204_214del11 (p.Gly69Argfs*10) mutation. The hiPSC lines retained the disease-causing mutation and exhibited stable pluripotent characteristics, providing a genetically relevant human cellular model for MPAN (Krogulec et al., 2024). These patient-specific hiPSC lines complement previous fibroblast-based studies of C19orf12 deficiency (Wydrych et al., 2025) and (Angelini et al., 2023) and provide a valuable platform for future mechanistic investigations in human neurons and glial cells.

To establish a human cellular model of fatty acid hydroxylase-associated neurodegeneration (FAHN), Efendic et al. generated a patient-derived hiPSC line (AKOSi010-A) from an individual carrying compound heterozygous FA2H pathogenic variants (p.Gly45Arg and p.His319Arg). The hiPSC line retained the patient-specific genetic background and pluripotent characteristics, providing the first disease-specific human stem cell model for FAHN and a platform for investigating the cellular mechanisms underlying FA2H deficiency (Efendic et al., 2022). Using the AKOSi010-A hiPSC line, Efendic et al. established a patient-derived neuron–oligodendrocyte co-culture model to investigate the effects of FA2H deficiency on myelination and autophagy. Differentiation into neurons, astrocytes, oligodendrocytes, and myelinating co-cultures showed that FA2H deficiency did not impair early neural lineage specification but was associated with reduced FA2H protein expression, abnormal intracellular localization, and impaired oligodendrocyte maturation. FA2H-deficient cultures exhibited reduced expression and altered organization of the major myelin proteins MBP and PLP, accompanied by decreased myelination, fewer nodes of Ranvier, longer internodes, and abnormal nodal morphology. In addition, impaired autophagic flux, characterized by altered LC3B and p62 expression and defective autophagosome–lysosome fusion, was observed, suggesting that defects in myelin maintenance and autophagy contribute to FAHN pathogenesis (Efendic et al., 2025).

In neuroferritinopathy, Moro et al. investigated how intracellular iron overload contributes to astrocyte dysfunction by generating hiPSC-derived astrocytes from patients carrying FTL1 pathogenic variants (469_484dup and 351delG_InsTTT) and comparing them with healthy and isogenic control astrocytes. Differentiated disease astrocytes showed a disease-relevant iron dyshomeostasis phenotype with significantly increased ferritin heavy chain (FtH) and ferroportin (Fpn) expression, reduced TfR1 and DMT1 levels, and progressive intracellular iron accumulation demonstrated by Perls staining, indicating excessive cytosolic iron retention. Iron-overloaded astrocytes acquired a transient reactive phenotype, with elongated stellate morphology, marked increases in extracellular IL 6, IL 1β, and glutamate release, and altered astrocyte reactivity markers, including reduced NRF2, increased LCN2, elevated IL 6 and iNOS transcripts, while EAAT2 expression remained largely unchanged, indicating that iron overload directly promotes astrocyte activation without impairing astrocyte differentiation. This reactive phenotype diminished by day 60 of differentiation, when inflammatory cytokine levels fell below control values, suggesting a transition from early activation to subsequent dysfunction. Mechanistically, Moro et al. showed that disease astrocytes exhibited progressively elevated total iron content and cytosolic labile iron pools, whereas mitochondrial labile iron remained largely unaffected, indicating that cytosolic redox-active iron is the principal driver of pathology. Persistent iron accumulation was associated with marked cellular senescence, evidenced by increased SA β galactosidase activity and elevated p62, together with activation of ferroptosis, demonstrated by increased MDA and progressive reduction of GPX4, indicating enhanced lipid peroxidation and impaired antioxidant defense. They concluded that intracellular free iron is sufficient to initiate astrocyte reactivity and that sustained iron overload subsequently drives senescence and ferroptotic cell death, establishing iron as a primary regulator of astrocyte-mediated neuroinflammation in neuroferritinopathy and highlighting brain-permeable iron chelation as a potential therapeutic approach (Moro et al., 2025).

Complementing these astrocytes based findings, Rohani et al. reported a familial case of FTH1-related neuroferritinopathy (NBIA9) by identifying a heterozygous frameshift variant c.465_466insG (p.Leu156Valfs17) in FTH1 in two affected Iranian brothers using exome sequencing and Sanger confirmation, and reviewed all reported FTH1 variants. They identified seven disease-associated FTH1 variants, four linked to neuroferritinopathy, showing that all confirmed neuroferritinopathy-causing variants are frameshift/truncating pathogenic variants located within the last exon. Unlike previously reported FTH1-related neuroferritinopathy cases, which predominantly presented during infancy with developmental delay and rapidly progressive neurological impairment, the brothers studied by Rohani had normal neurodevelopment and late disease onset at 13 and 26 years, thereby expanding the recognized phenotypic spectrum of NBIA9. Clinically, both developed dystonia, dysarthria, dysphagia, dystonic tremor, and movement abnormalities; the older brother additionally exhibited gait imbalance, occasional choking episodes, and mild cognitive impairment, while the younger showed mild hypokinesia without cognitive decline. MRI demonstrated iron deposition within the basal ganglia, including striatum, globus pallidus, substantia nigra, thalamus, and dentate nucleus, with T1 and T2 signal abnormalities; CT showed no calcification. Serum iron and ceruloplasmin were normal in both, whereas serum ferritin was elevated only in the older brother and normal in the younger, indicating that systemic iron indices may not reliably reflect brain iron accumulation. Based on comparison with earlier cases, Rohani et al. proposed that the location of truncating variants within the C-terminal E helix of FTH1 may influence disease severity and age at onset by altering ferritin assembly and function through a dominant negative mechanism, while emphasizing that this remains speculative in the absence of functional studies (Rohani et al., 2026). In combination, (Moro et al., 2025) and (Rohani et al., 2026) link FTL1/FTH1 pathogenic variants to astrocyte-mediated iron overload, reactive and senescent phenotypes, ferroptosis markers, and adolescent/adult onset neuroferritinopathy with widespread basal ganglia iron deposition.

Overall, these studies indicate that, beyond PKAN and PLAN, NBIA subtypes such as MPAN, FAHN, and neuroferritinopathy can be reproduced in patient‑derived fibroblasts and hiPSC‑based cellular models, and that in these systems the predominant abnormalities involve mitochondrial bioenergetics and metabolic adaptation in MPAN, myelin protein organization together with autophagic flux in FAHN, and astrocyte iron handling, senescence, and ferroptosis in neuroferritinopathy. In MPAN, the combination of recessive and dominant C19orf12 studies shows consistent mitochondrial and metabolic disturbances, with additional iron‑storage and autophagy changes in autosomal dominant and mosaic cases; in FAHN, the two hiPSC‑derived models link FA2H pathogenic variants to defective axonal myelination and impaired autophagosome–lysosome fusion; and in neuroferritinopathy, astrocyte models and familial clinical data together associate FTL1/FTH1 pathogenic variants with progressive cytosolic iron overload, reactive and senescent astrocyte phenotypes, and adolescent or adult‑onset basal ganglia iron deposition. Important limitations include modest patient numbers in each series, reliance on single cell types rather than integrated brain circuits, and incomplete functional testing of some proposed genotype–phenotype mechanisms, particularly for FTH1 truncating variants, where dominant‑negative effects remain hypothetical. Even within these constraints, the available evidence supports further work using patient‑specific iPSC‑derived neurons, astrocytes, and oligodendrocytes, and motivates exploration of targeted strategies aimed at improving mitochondrial function in MPAN, stabilizing myelin and autophagy in FAHN, and modulating glial iron–lipid homeostasis and ferroptotic vulnerability in neuroferritinopathy and related NBIA subtypes.

## Human Cellular Models and iPSC‑Derived Systems Across NBIA

Human cellular and iPSC-derived models now provide disease-relevant platforms for dissecting NBIA mechanisms across BPAN, PARK9/ATP13A2-associated disease, and PKAN, and for testing candidate therapies in patient-specific cells, as highlighted in **Table 5.** Recent studies illustrate how iPSC-derived neurons, astrocytes, oligodendrocytes, and microglia capture autophagy–lysosome defects, CoA metabolism–linked iron dyshomeostasis, and neuroimmune changes, and how these models can be used for mechanistic and pharmacological work.

**Table 5.** Recent advances in human cellular and iPSC-derived models of NBIA.

Reference	NBIA subtype	Human cellular model	Main findings
(Ripamonti et al., 2022)	PKAN	hiPSC-derived astrocytes	Demonstrated impaired endosomal trafficking, increased transferrin uptake, and iron accumulation; CoA and 4-PBA partially restored trafficking defects.
(Santambrogio et al., 2022)	PKAN	hiPSC-derived neurons and astrocytes	Established the first human PKAN neuronal–astrocyte model showing mitochondrial dysfunction, ferroptosis, iron accumulation, and astrocyte-mediated neurotoxicity.
(Santambrogio et al., 2023)	PKAN	hiPSC-derived astrocytes	Leriglitazone improved mitochondrial function, cell viability, and reduced iron accumulation.
(Santambrogio et al., 2024)	PKAN	hiPSC-derived astrocytes	Showed that mitochondrial iron deficiency precedes secondary cytosolic iron accumulation through defective vesicular trafficking.
(Tournois et al., 2026)	BPAN	Patient-derived hiPSC lines	Generated and characterized six BPAN hiPSC lines as a resource for disease modeling and therapeutic studies.
(Özata et al., 2026)	BPAN	iPSC-derived microglia	Demonstrated altered microglial homeostasis, lysosomal dysfunction, autophagy defects, and immune remodeling.
(Tsukiboshi et al., 2026)	ATP13A2/ PARK9	iPSC-derived dopaminergic neurons	Recapitulated lysosomal dysfunction and established a platform for therapeutic compound screening.

Özata et al. established the first iPSC-derived microglia model from BPAN patients carrying WDR45 pathogenic variants to investigate the contribution of microglia to BPAN pathophysiology, which had remained largely unexplored despite BPAN sharing iron accumulation and dopaminergic neuron degeneration features with Parkinson’s disease. BPAN iPSC-derived microglia expressed canonical microglial markers (IBA1, TMEM119, P2RY12), maintained inflammatory responsiveness to lipopolysaccharide, and preserved phagocytic uptake of human neuromelanin, confirming successful differentiation into functionally competent microglia. Targeted NanoString transcriptomics showed that BPAN microglia shifted from a homeostatic state toward a stress adapted, transcriptionally reprogrammed phenotype with reduced homeostatic genes (CX3CR1, MEF2C) and increased inflammatory and stress associated genes (CD44, CST7, VEGFA, PTGER3), dysregulated autophagy pathways, remodeling of NF κB and cytokine signaling, altered immune regulatory circuits, reduced interferon responsive gene expression, and impaired DNA repair and cell cycle pathways, consistent with chronic cellular stress and enhanced survival signaling rather than acute inflammatory cell death. hiSPECS secretome profiling revealed reduced secretion of multiple lysosomal enzymes and hydrolases (ARSA, IDUA, CTSA, CTSV, CTSD, GLB1, GBA1, NEU1) and decreased osteopontin (SPP1), with increased immune-associated surface proteins (CD1A, CD74, MICA, MMP12), indicating altered lysosomal trafficking, impaired degradative and phagocytic functions, and remodeled antigen presentation-related signaling. Integration of transcriptomic and secretomic data demonstrated concordant RNA and protein changes, and Özata et al. concluded that BPAN microglia display a distinct immunomodulatory and secretory phenotype characterized by disrupted microglial homeostasis, altered autophagy–lysosomal pathways, selective immune signaling remodeling, and chronic stress-associated reprogramming, suggesting microglial dysfunction as a contributor to BPAN pathogenesis and a potential neuroimmune therapeutic target (Özata et al., 2026).

To provide a human cellular model of β-propeller protein-associated neurodegeneration (BPAN), Tournois et al. generated six patient-derived hiPSC lines from four unrelated individuals carrying distinct WDR45 pathogenic variants (c.2T > A, c.19C > T, c.400C > T, and c.81_82del). These hiPSC lines retained the patient-specific mutations and exhibited stable pluripotent characteristics, providing a genetically diverse human stem cell resource that captures both mutation- and sex-specific variability in BPAN (Tournois et al., 2026). This collection complements recent human microglial studies (Özata et al., 2026) and provides a valuable platform for investigating WDR45 function, autophagy-related mechanisms, and therapeutic strategies across multiple neural cell types.

Tsukiboshi et al. developed a patient-specific iPSC-derived dopaminergic neuron model of PARK9 (ATP13A2-associated Parkinson’s disease) and an isogenic CRISPR/Cas9 mutation corrected control to investigate autophagy–lysosome dysfunction and enable compound screening. PARK9 neurons recapitulated disease-associated abnormalities: impaired lysosomal acidification, reduced mature cathepsin D, accumulation of CD63-positive endolysosomal vesicles, increased LC3B-positive autophagosomes and p62, cytoplasmic accumulation of phosphorylated α synuclein (pSer129), and elevated cleaved caspase 3, indicating increased vulnerability, whereas ATP13A2 correction largely ameliorated these phenotypes, confirming their dependence on ATP13A2 dysfunction. Using these neurons, Tsukiboshi et al. set up a high content imaging assay based on LC3B-positive autophagosome accumulation and screened a 320-compound pharmacological inhibitor library in three steps, identifying 19 candidates that reduced autophagosome accumulation through mechanisms consistent with improved lysosome-dependent autophagic processing. Further validation showed several compounds reproducibly decreased LC3B-positive autophagosomes and cleaved caspase 3, with paroxetine, Ro 25 6981, amisulpride, and PK11195 selected for detailed evaluation because of their favorable effects. These compounds showed compound dependent improvements in lysosomal acidification, reduced CD63 positive vesicle accumulation, decreased cytoplasmic pSer129 α synuclein, and reduced cleaved caspase 3, although effect size varied, leading the authors to conclude that PARK9 iPSC derived dopaminergic neurons are a robust human model of lysosomal dysfunction–associated Parkinson’s disease and a practical platform for identifying candidate autophagy–lysosome targeting therapies (Tsukiboshi et al., 2026).

Ripamonti et al. examined PKAN pathomechanisms using patient-derived hiPSC astrocytes carrying PANK2 pathogenic variants to determine how impaired CoA biosynthesis contributes to brain iron accumulation. PKAN astrocytes showed significantly increased transferrin uptake despite unchanged TfR1 expression, indicating dysregulation of transferrin receptor–mediated iron internalization. Using the SynaptoZip–Synbond biosensor system, the authors found profound impairment in constitutive endosomal trafficking, with enhanced vesicular activity, defective endosomal recruitment, abnormal vesicle recycling, reduced diffusion coefficients, shorter intracellular transport distances, and altered endosomal dynamics compared with controls, suggesting that defective CoA biosynthesis disrupts membrane fusion and intracellular vesicular trafficking and thereby promotes excessive transferrin-mediated iron uptake and intracellular iron accumulation. Therapeutically, treatment with CoA and 4-phenylbutyric acid (4 PBA) partially restored endosomal trafficking, normalized vesicular dynamics, reduced transferrin uptake, and improved trafficking defects, with 4 PBA showing particularly robust rescue of several endosomal abnormalities. Ripamonti et al. concluded that PKAN hiPSC astrocytes are a clinically relevant human model demonstrating that defective CoA metabolism impairs endosomal trafficking and promotes iron overload, and that modulation of intracellular vesicular trafficking represents a promising target for PKAN and related NBIA disorders (Ripamonti et al., 2022).

Santambrogio et al. created the first hiPSC-derived neuronal and astrocyte model that recapitulates hallmark PKAN features, aiming to elucidate how PANK2 deficiency leads to massive brain iron accumulation. Patient hiPSCs were differentiated into striatal-like medium spiny neurons (d MSNs) and astrocytes; both showed pronounced cytosolic iron deposition by Perls staining, with astrocytes carrying the highest burden (∼50% Perls positive cells) and neurons showing reduced viability. PKAN astrocytes exhibited swollen mitochondria with disrupted cristae, significantly impaired respiratory activity, altered iron metabolism (increased ferritin and ferroportin, reduced TfR1, DMT1, and NCOA4), elevated oxidative stress, and ferroptosis activation, together with a reactive stellate phenotype. Astrocytes released significantly higher glutamate, inducing neurotoxicity and reducing the survival of co-cultured PKAN glutamatergic neurons, highlighting astrocyte-mediated excitotoxicity. CoA supplementation significantly reduced iron accumulation, improved mitochondrial function, decreased oxidative and ferroptotic markers, and partially rescued neuronal viability, leading Santambrogio et al. to conclude that mitochondrial dysfunction, cytosolic iron overload, ferroptosis, and astrocyte-mediated neurotoxicity collectively drive PKAN pathogenesis and that CoA supplementation is a promising therapeutic strategy (Santambrogio et al., 2022).

Building on this model, Santambrogio et al. tested leriglitazone, a brain penetrant PPARγ agonist, in PKAN hiPSC astrocytes to determine whether enhancing mitochondrial function could reverse PANK2-related defects. PKAN astrocytes in this study again showed reduced viability, impaired mitochondrial respiration, and marked cytosolic iron accumulation; treatment with 100 nM leriglitazone did not alter differentiation but significantly improved cell viability and restored respiration, in several measures outperforming CoA supplementation. Leriglitazone also markedly reduced intracellular iron, decreasing iron-positive astrocytes by 78–91% versus 70–83% with CoA, suggesting superior prevention of pathological iron deposition. The authors proposed that PPARγ activation enhances mitochondrial biogenesis and respiratory function while reducing oxidative stress and neuroinflammation, thereby restoring cellular energy metabolism and indirectly limiting iron accumulation linked to mitochondrial dysfunction. Santambrogio et al. concluded that leriglitazone effectively rescues mitochondrial dysfunction, improves astrocyte survival, and alleviates iron overload in patient-derived PKAN astrocytes, highlighting PPARγ activation as a promising disease-modifying strategy for PKAN and possibly other NBIA forms (Santambrogio et al., 2023).

In a related mechanistic study, Santambrogio et al. used PKAN hiPSC astrocytes to examine how CoA deficiency disrupts intracellular iron trafficking and leads to iron deposition. They found significantly reduced α-tubulin acetylation and increased β-tubulin phosphorylation, causing defective microtubule dynamics and impaired vesicular trafficking, and super-resolution microscopy showed a marked reduction in transferrin–loaded endosome–mitochondria interactions, limiting delivery of transferrin-bound iron to mitochondria. As a result, PKAN astrocytes developed mitochondrial iron deficiency despite cytosolic iron overload, evidenced by increased cytosolic labile iron and total iron with Perls positive deposits. Reduced mitochondrial iron impaired heme synthesis and iron–sulfur (ISC) formation, leading to decreased mitochondrial and cytosolic aconitase activities and disrupted respiratory function. Mechanistically, the authors proposed that mitochondrial iron deficiency activates compensatory iron uptake via dysregulated iron-responsive proteins, driving continuous iron influx, ferritin accumulation, and progressive cytosolic iron deposition, and concluded that mitochondrial iron deficiency is the initiating event driving secondary cytosolic iron overload in PKAN rather than iron accumulation being primary. They further suggested that restoring CoA metabolism or enhancing microtubule acetylation to normalize mitochondrial iron delivery may be promising strategies for PKAN and other NBIA disorders (Santambrogio et al., 2024).

**Figure 1.**
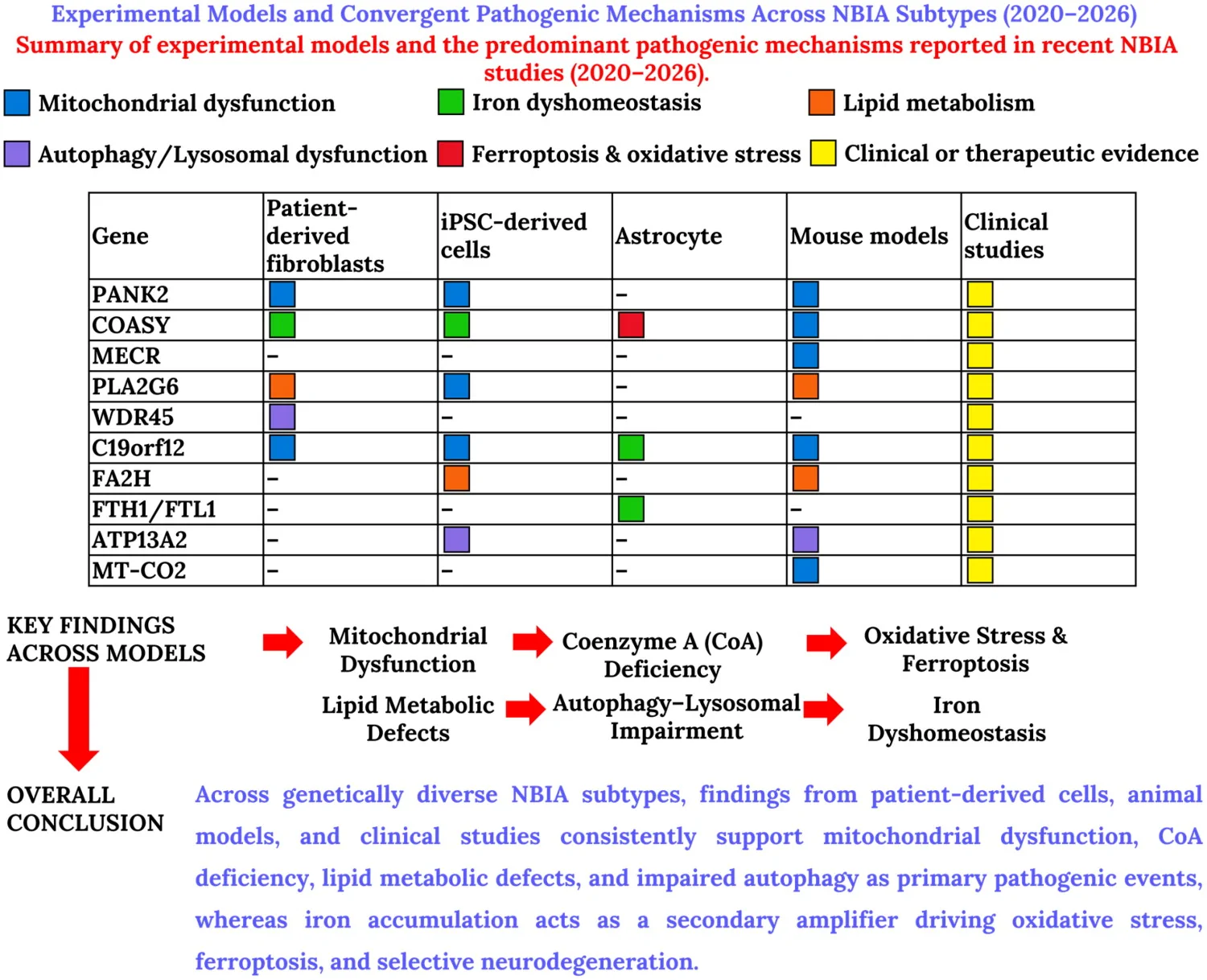
Experimental models and convergent pathogenic mechanisms across NBIA subtypes (2020–2026). Summary of the major experimental models and predominant pathogenic mechanisms reported across major NBIA subtypes in recent studies. The figure integrates findings from patient-derived fibroblasts, iPSC-derived cells, astrocytes, mouse models, and clinical studies, illustrating the convergence of genetically distinct NBIA subtypes on shared pathogenic processes. Each colored square represents the predominant pathogenic mechanism reported in the corresponding experimental model for that NBIA subtype.

Taken together, (Özata et al., 2026) and (Tournois et al., 2026) show that BPAN can be modeled both in iPSC-derived microglia, which reveal a stress-adapted immunomodulatory and lysosomal secretory phenotype, and in a mutation-diverse panel of pluripotent lines that provide the basis for deriving multiple neural cell types with defined WDR45 variants. (Tsukiboshi et al., 2026) extend this approach to PARK9/ATP13A2-associated disease, demonstrating that patient-derived iPSC dopaminergic neurons faithfully recapitulate autophagy–lysosome dysfunction and can be used in quantitative high content screens to identify compounds that normalize autophagosome turnover, lysosomal acidification, and α-synuclein handling. In parallel, (Ripamonti et al., 2022) and (Santambrogio et al., 2022, 2023, 2024) collectively establish PKAN hiPSC astrocytes and neurons as human models in which impaired CoA metabolism produces mitochondrial failure, mitochondrial iron deficiency with secondary cytosolic iron overload, ferroptosis, astrocyte-mediated excitotoxicity, and vesicular trafficking defects, and in which CoA, 4 PBA, and leriglitazone can partially or substantially rescue endosomal dynamics, mitochondrial respiration, and iron dyshomeostasis. Important limitations across these studies include relatively small numbers of patients and clones, the predominant focus on single cell types rather than integrated neural circuits or organoids, and the lack of *in vivo* confirmation for most candidate therapies identified *in vitro*. Even so, the current human cellular and iPSC derived NBIA models provide a mechanistically coherent framework for linking specific gene defects (WDR45, ATP13A2, PANK2) to defined cellular pathologies and support a roadmap in which future work uses patient specific neurons, astrocytes, oligodendrocytes, and microglia and potentially 3D or co culture systems to refine disease mechanisms, validate pharmacological hits, and design early phase therapeutic studies grounded in human disease biology. Collectively, findings from patient-derived fibroblasts, iPSC-derived cells, astrocytes, animal models, and clinical studies demonstrate that genetically diverse NBIA subtypes converge on common pathogenic mechanisms, including mitochondrial dysfunction, CoA deficiency, lipid metabolic defects, autophagy–lysosomal impairment, iron dyshomeostasis, and ferroptosis, as highlighted in **Figure 1.**

## Iron Localization, Neurotransmitter Metabolism and Imaging Biomarkers

**Figure 2.**
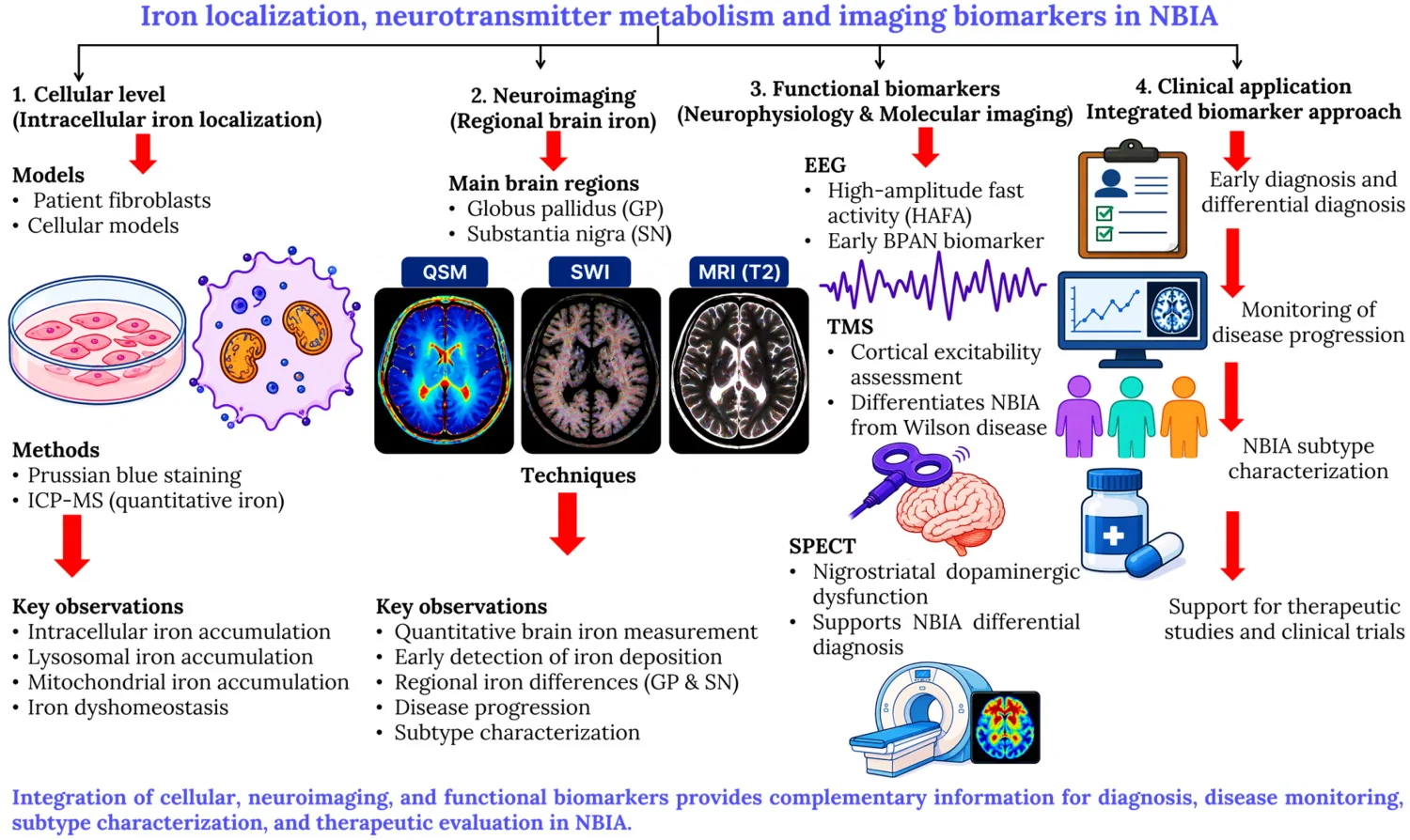
Multilevel approaches for evaluating iron localization, neurotransmitter metabolism, and imaging biomarkers in neurodegeneration with brain iron accumulation (NBIA).

Iron localization, neurotransmitter metabolism, and imaging biomarkers are increasingly used to refine diagnosis, understand metal-related neurotoxicity, and distinguish NBIA from overlapping disorders, particularly in conditions where conventional MRI may underestimate iron deposition or structural change, as highlighted in **Table 6.** Recent studies together show how iron can be quantified at the cellular and tissue level, how cortical excitability signatures differ between iron and copper overload, and how advanced MRI and functional imaging can reveal early or atypical NBIA-like patterns. **Figure 2** summarizes the complementary approaches currently used to evaluate iron localization, neurotransmitter dysfunction, and imaging biomarkers in NBIA, integrating evidence from cellular studies, neuroimaging, functional biomarkers, and their clinical applications.

**Table 6.** Recent studies on iron localization, neurotransmitter metabolism, and imaging biomarkers in NBIA.

Reference	NBIA subtype / disorder	Biomarker / model	Main findings
(Kidokoro et al., 2020)	BPAN	EEG and MRI	Identified high-amplitude fast activity (HAFA) on EEG as an early biomarker preceding characteristic MRI iron deposition.
(Dubbioso et al., 2020)	Kufor–Rakeb syndrome, aceruloplasminemia	Transcranial magnetic stimulation (TMS)	Demonstrated distinct cortical excitability patterns associated with altered GABAergic neurotransmission in NBIA.
(Kimura et al., 2021)	BPAN	Serial MRI (SWI/QSM)	Showed that SWI and QSM detect early brain iron accumulation before conventional MRI changes.
(Leclert et al., 2024)	SLC6A1-related disorder	MRI and dopamine transporter SPECT	Reported pallidal iron accumulation and dopaminergic denervation, expanding the differential diagnosis of NBIA.
(Uygun et al., 2025)	PKAN, MPAN, PLAN, KRS	Quantitative susceptibility mapping (QSM)	Demonstrated subtype-specific regional iron accumulation and supported QSM as a quantitative imaging biomarker.
(Erterek et al., 2026)	Kufor–Rakeb syndrome (ATP13A2)	Patient fibroblasts and cellular iron assays	Showed mutation-dependent intracellular iron accumulation, suggesting that cellular iron analysis may complement MRI.
(Murakami et al., 2026)	PARK9 / Kufor–Rakeb syndrome	ATP13A2 knockdown cell model	Demonstrated that lysosomal dysfunction, impaired mitophagy, and defective heme synthesis promote intracellular iron accumulation.

Erterek et al. investigated whether different ATP13A2 pathogenic variants associated with Kufor–Rakeb syndrome (KRS) produce different levels of intracellular iron accumulation, addressing the debate over whether KRS should be considered an NBIA disorder, given that conventional MRI does not consistently show iron deposition. They clinically evaluated three unrelated KRS families and identified two previously unreported ATP13A2 variants, a homozygous in-frame deletion (p.Leu518_Thr519del) and a homozygous missense mutation (p.Leu939Pro) alongside a previously reported frameshift mutation (p.Pro474fs), confirmed by exome sequencing and segregation analysis. Using patient-derived fibroblasts and MCF7 cells overexpressing wild-type or mutant ATP13A2, they assessed intracellular iron by Prussian Blue staining and ICP MS and evaluated cell viability with MTT assays. All three pathogenic variants caused intracellular iron accumulation, but the magnitude depended on mutation type, with frameshift and deletion variants producing significantly greater iron accumulation than the missense variant, consistently across fibroblasts and engineered cells, and quantitatively confirmed by ICP MS. They further showed that the frameshift mutation reduced ATP13A2 mRNA expression, whereas deletion and missense pathogenic variants did not alter transcript levels, indicating distinct molecular consequences among mutation classes. Under iron treatment, mutation-bearing cells showed reduced viability, with missense mutation cells less sensitive than frameshift or deletion cells, and overexpression of wild-type ATP13A2 partially restored fibroblast viability under iron stress, supporting a protective role for functional ATP13A2 against iron-induced cytotoxicity. Overall, Erterek et al. concluded that different ATP13A2 pathogenic variants lead to graded intracellular iron accumulation and toxicity and that cellular iron analysis may be more sensitive than MRI for detecting mutation-dependent iron overload in KRS, helping explain inconsistent imaging reports (Erterek et al., 2026).

Complementing this mutation-level cellular analysis, Murakami et al. investigated how ATP13A2 deficiency disrupts intracellular iron homeostasis mechanistically in a PARK9/Kufor–Rakeb model using ATP13A2 knockdown SH SY5Y cells overexpressing α-synuclein. They demonstrated lysosomal dysfunction (increased lysosomal pH, reduced autophagic flux, accumulation of LC3 II and p62, increased intracellular α synuclein), confirming impaired lysosomal degradation and autophagy. ATP13A2 deficiency increased total intracellular iron and labile Fe^2+^, with organelle-specific probes revealing iron accumulation in both mitochondria and lysosomes, accompanied by increased ferritin, elevated mitochondrial ROS, reduced viability, and partial rescue by the iron chelator deferoxamine and the ferroptosis inhibitor Ferrostatin 1. Mechanistically, ATP13A2 knockdown upregulated TfR and DMT1 mRNA, increased TfR protein without changing IRP2 levels, indicating that iron uptake remained elevated despite intracellular iron overload. Blocking iron uptake using apo transferrin or the DMT1 inhibitor glibenclamide reduced intracellular iron and mitochondrial oxidative stress and improved survival, supporting the role of excessive iron influx in cytotoxicity. Murakami et al. further showed impaired mitophagy, reduced mitochondrial respiration, ATP production, spare capacity, and heme synthesis; similar heme reduction was seen in PINK1-deficient cells. Because heme is needed for IRP2 degradation, reduced heme prevented appropriate IRP2-mediated regulation of iron transporters, maintaining persistent iron uptake despite overload and establishing a cycle of mitochondrial dysfunction and progressive iron accumulation. Together, (Erterek et al., 2026) and (Murakami et al., 2026) link ATP13A2 pathogenic variants and deficiency to mutation-dependent iron loading, lysosomal–mitochondrial dysfunction, and dysregulated iron transport, providing both quantitative and mechanistic explanations for iron dyshomeostasis in KRS/PARK9 (Murakami et al., 2026).

At the systems level, Uygun et al. quantitatively evaluated brain iron accumulation in genetically confirmed NBIA disorders using quantitative susceptibility mapping (QSM) to define subtype-specific patterns and assess QSM as a diagnostic and monitoring biomarker. They analyzed 16 NBIA patients (including PKAN, MPAN, PLAN, and Kufor–Rakeb syndrome) and 11 matched controls with susceptibility weighted imaging at 3 T, delineating basal ganglia regions and deriving QSM-based susceptibility values. Iron accumulation was significantly increased in the globus pallidus (GP) of NBIA patients versus controls (p = 0.008); PKAN patients had about 2.5-fold higher GP iron (p = 0.001), and MPAN patients showed ∼2.5-fold greater iron in both GP (p = 0.005) and substantia nigra (SN) (p = 0.036). ROC analysis identified a GP susceptibility >0.1133 ppm as a threshold increasing PKAN likelihood ≈18-fold, with 75% sensitivity and 82% specificity for distinguishing PKAN from controls. Within PKAN, atypical cases had ∼2.5-fold higher SN iron than classic PKAN (p = 0.024), and compound heterozygous PKAN patients had fourfold higher caudate iron than homozygous patients (p = 0.036), indicating genotype and phenotype-dependent regional differences. Putaminal iron was significantly lower in NBIA patients than in controls (p = 0.015), and age independently influenced susceptibility in several deep gray structures, emphasizing age adjustment when interpreting QSM. Although PLAN and KRS numbers were small, both groups showed elevated iron compared with controls. Uygun et al. concluded that QSM is a sensitive, non-invasive method for quantitatively detecting regional iron in NBIA, with the GP consistently showing the greatest burden, supporting QSM as a potential imaging biomarker for early diagnosis, longitudinal monitoring, and therapeutic trials (Uygun et al., 2025).

Focusing on BPAN, Kimura et al. investigated serial brain MRI changes in pediatric WDR45-related BPAN to identify early imaging features that could aid diagnosis before classical adult MRI patterns appear. They retrospectively evaluated 21 MRIs from four genetically confirmed BPAN patients aged 1–7 years and performed QSM of GP, SN, and deep cerebellar nuclei (DCN), comparing with 10 age-matched disease controls. Only one of four patients was suspected of BPAN based on imaging before genetic confirmation, highlighting diagnostic difficulty in early childhood. Serial MRI showed no GP or SN abnormalities initially; T2 hypointensity appeared between 4 and 7 years, whereas SWI hypointensity appeared earlier (2–7 years), indicating SWI’s greater sensitivity for early iron deposition. Three patients had persistent bilateral DCN T2 hyperintensity, and three developed transient T2 hyperintensity and swelling of GP, SN, and/or DCN during fever or seizures, which improved subsequently. Additional findings included progressive cerebral and cerebellar atrophy, corpus callosum thinning in all patients, and delayed myelination that eventually completed, suggesting supportive structural markers of pediatric BPAN. QSM showed significantly higher susceptibility in GP and SN in BPAN versus controls (P = 0.005 for both), with no significant DCN difference, confirming selective basal ganglia iron. Kimura et al. concluded that serial MRI, especially SWI and QSM, can detect early and progressive brain iron accumulation before conventional findings become evident, and that early SWI hypointensity of GP/SN, persistent DCN T2 hyperintensity, callosal thinning, transient swelling during pyrexia or seizures, delayed myelination, and progressive atrophy should prompt early WDR45 testing (Kimura et al., 2021).

In BPAN, Kidokoro et al. asked whether high amplitude fast activity (HAFA) on EEG could serve as an early diagnostic biomarker, given that characteristic MRI iron deposition often appears later in childhood. They retrospectively analyzed childhood EEGs from five genetically confirmed BPAN patients with WDR45 pathogenic variants and compared them with 143 EEGs from 59 children with other neurological conditions. All five BPAN patients had their first EEG between 12 and 21 months; each showed diffuse, continuous HAFA (20–50 Hz; >50 μV) during wakefulness and sleep, with four lacking a clear posterior dominant rhythm while awake. In contrast, although excessive fast activity was seen in 28% of comparison EEGs, only two recordings (1.4%) showed HAFA exceeding 50 μV, both mainly during sleep in children on antiepileptics, and their awake EEGs retained a normal posterior dominant rhythm, distinguishing them from the BPAN pattern. Early MRI in BPAN showed mostly delayed myelination, with GP/SN iron deposition appearing later; patients commonly had elevated AST, LDH, and NSE in early childhood. Kidokoro et al. concluded that diffuse continuous HAFA on EEG during both wake and sleep is an important early clue to BPAN, especially in children not receiving benzodiazepines or barbiturates, and that recognizing this pattern with supportive lab and MRI findings should prompt early WDR45 genetic testing (Kidokoro et al., 2020).

To compare cortical neurotransmission in iron versus copper overload, Dubbioso et al. used transcranial magnetic stimulation (TMS) to evaluate glutamatergic and GABAergic circuits in hereditary NBIA and Wilson’s disease (WD). They studied three NBIA patients (two ATP13A2-related PARK9/KRS, one aceruloplasminemia) and seven neurological WD patients, measuring resting and active motor thresholds (RMT, AMT), short interval intracortical inhibition (SICI), intracortical facilitation (ICF), short latency afferent inhibition (SAI), and cortical silent period (CSP). WD patients had significantly higher RMT and AMT, reduced SICI, and shortened CSP, indicating reduced motor cortex excitability and impairment of both GABA(A) and GABA(B) mediated inhibitory circuits. By contrast, NBIA patients showed normal motor thresholds, preserved SICI, ICF, and SAI, but significantly prolonged CSP, suggesting selective dysfunction of GABA(B) mediated intracortical inhibition rather than broad excitability changes. The aceruloplasminemia patient had extensive iron deposition (putamen, caudate, posterior thalami, red nuclei, SN, dentate nuclei, cerebral/cerebellar cortex) on SWI and cerebellar/posterior putamen hypometabolism on FDG PET with mild executive deficits, despite largely preserved electrophysiology. Notably, in ATP13A2-related KRS patients, TMS abnormalities were present even when MRI had not yet demonstrated iron accumulation, suggesting that changes in cortical excitability may precede structural iron detection. Dubbioso et al. concluded that iron and copper overload produce distinct cortical excitability signatures, with prolonged CSP characterizing NBIA and higher motor thresholds plus reduced SICI and shortened CSP characterizing WD, highlighting TMS as a physiological biomarker for differentiating hereditary metal accumulation disorders and probing iron/copper effects on human cortical neurotransmission (Dubbioso et al., 2020).

Leclert et al. reported a 26-year-old woman with a de novo pathogenic SLC6A1 variant who developed Parkinsonism with bilateral globus pallidus iron deposition and presynaptic dopaminergic denervation, expanding the clinical and radiological spectrum of SLC6A1-related disorders and suggesting inclusion in the NBIA differential. The patient had developmental delay, mild intellectual disability, behavioral and psychiatric symptoms, hallucinations, and hyperkinetic movements; after prolonged neuroleptic treatment, she developed akinetic rigid parkinsonism with mild bilateral hand dystonia, choreic hand movements, hypophonia, gait impairment, and balance disturbance, with minimal levodopa response. Neuropsychology showed mild intellectual disability (IQ 52) with visuospatial, executive, and attention deficits; routine labs, including iron and copper studies, were unremarkable. I^12^³ FP β CIT SPECT revealed bilateral striatal presynaptic dopaminergic denervation, while 3 T MRI with SWI showed bilateral GP lesions with iron accumulation and milder involvement of caudate and SN; putamen, thalami, and cortex were spared, and CT excluded calcification. Whole genome sequencing identified a de novo missense SLC6A1 variant (c.187G > A); no pathogenic variants were found in NBIA genes or others explaining the imaging phenotype. Leclert et al. noted that previous SLC6A1-related disorders typically had normal or nonspecific MRI and no dopaminergic degeneration, whereas this case showed NBIA-like MRI and nigrostriatal dysfunction; they concluded that SLC6A1-related disorders can present with brain iron accumulation and dopaminergic neurodegeneration and should be considered among NBIA differentials, especially in patients with neurodevelopmental, psychiatric, and movement symptoms plus pallidal iron (Leclert et al., 2024).

Taken together, (Erterek et al., 2026) and (Murakami et al., 2026) show that ATP13A2 related Kufor–Rakeb syndrome/PARK9 is characterized both by mutation-dependent gradients of intracellular iron loading and by a mechanistic cascade in which lysosomal dysfunction leads to impaired autophagic flux, defective mitophagy, reduced mitochondrial respiration and heme synthesis, dysregulated IRP2-mediated sensing of iron transporters, and persistent iron influx that drives progressive iron accumulation and ferroptotic vulnerability. (Uygun et al., 2025) and (Kimura et al., 2021) together demonstrate that advanced MRI techniques, quantitative susceptibility mapping (QSM), susceptibility weighted imaging (SWI), and serial conventional MRI, can sensitively quantify regional iron deposition and reveal early structural changes across NBIA subtypes, with the globus pallidus and substantia nigra emerging as consistently involved regions and with genotype and phenotype dependent differences in iron burden (for example, classic versus atypical PKAN, or MPAN versus other NBIA). In parallel, (Kidokoro et al., 2020) show that diffuse, continuous high amplitude fast activity (HAFA) on EEG during wakefulness and sleep provides an early functional biomarker for BPAN in toddlers before characteristic basal ganglia iron is visible on MRI, while (Dubbioso et al., 2020) reveal distinct transcranial magnetic stimulation (TMS) signatures that separate NBIA (selective prolongation of the cortical silent period with preserved motor thresholds and GABA(A) mediated inhibition) from Wilson’s disease (higher motor thresholds, reduced SICI, shortened CSP), highlighting cortical neurotransmission profiles as physiological markers of metal related neurodegeneration. Finally (Leclert et al., 2024) expand the phenotype of SLC6A1-related disorder by showing that it can present with pallidal iron accumulation on SWI, presynaptic nigrostriatal dopaminergic denervation on SPECT, and an akinetic rigid parkinsonian syndrome, even in the absence of pathogenic variants in known NBIA genes, thereby introducing SLC6A1 into the NBIA differential for patients with neurodevelopmental and psychiatric features. Important limitations across these studies include small sample sizes, heterogeneity of imaging and electrophysiological protocols, and the lack of large, longitudinal cohorts to validate each biomarker for routine diagnosis and monitoring, especially in rarer genotypes. Nonetheless, taken together, these cellular, physiological, and imaging data support an integrated roadmap in which quantitative iron imaging (QSM/SWI), electrophysiological measures (EEG HAFA patterns and TMS indices), and targeted cellular assays of iron handling are combined with careful clinical phenotyping and genetic testing to refine NBIA diagnosis, delineate subtype specific mechanisms of iron linked neurotransmitter dysfunction, track disease progression, and design future therapeutic trials targeting iron localization, cortical and basal ganglia neurotransmission, and mitochondrial–lysosomal cross talk in ATP13A2, WDR45, SLC6A1, and other NBIA associated disorders.

### Cellular and Subcellular Localization of Iron Accumulation in NBIA

Recent studies have extended the understanding of iron accumulation in NBIA beyond regional brain deposition by examining its localization at the cellular and subcellular levels. Rather than relying solely on conventional neuroimaging, these investigations have employed patient-derived cellular models and quantitative imaging approaches to evaluate intracellular iron distribution and its relationship with organelle dysfunction. Together, these studies indicate that iron accumulation is heterogeneous across NBIA subtypes and experimental models, emphasizing the importance of considering both intracellular and regional localization when interpreting disease mechanisms. Studies investigating ATP13A2**-**associated disease have provided direct evidence of intracellular iron accumulation using patient-derived cellular models. Erterek et al. examined primary fibroblasts from individuals carrying three distinct ATP13A2 mutations and quantified intracellular iron using Prussian blue staining and inductively coupled plasma mass spectrometry (ICP-MS). All three mutations were associated with intracellular iron accumulation, although the degree of accumulation differed among mutation types, with frameshift and deletion variants exhibiting greater iron accumulation than the missense variant. The authors further demonstrated that transient overexpression of wild-type ATP13A2 reduced iron-induced cell death, supporting a role for ATP13A2 in intracellular iron homeostasis. Importantly, this study proposed that assessment of iron accumulation at the cellular level may complement conventional MRI, particularly in patients in whom basal ganglia iron deposition is absent or inconclusive on neuroimaging (Erterek et al., 2026). Complementary mechanistic evidence was reported by Murakami et al. using an ATP13A2-deficient cellular model. The authors found that ATP13A2 suppression resulted in impaired lysosomal function together with iron accumulation within cellular organelles. They also reported mitochondrial dysfunction, impaired mitophagy, and reduced heme synthesis capacity, all of which were associated with disruption of intracellular iron homeostasis. Based on these findings, the study concluded that lysosome-associated mitochondrial dysfunction contributes to altered intracellular iron regulation in ATP13A2-deficient cells. Although this work was performed in a cellular model rather than patient tissue, it provides experimental evidence linking organelle dysfunction with intracellular iron accumulation (Murakami et al., 2026).

While cellular studies have focused on intracellular iron distribution, recent neuroimaging studies have characterized the anatomical localization of iron accumulation *in vivo.* Uygun et al. used quantitative susceptibility mapping (QSM) to measure iron content in genetically confirmed NBIA patients and reported significantly increased susceptibility values within the globus pallidus, with additional increases in the substantia nigra observed in MPAN patients. These findings demonstrate that quantitative MRI can identify regional differences in iron deposition among NBIA subtypes, complementing observations obtained from cellular studies (Uygun et al., 2025). Similarly, Kimura et al. evaluated serial MRI examinations in pediatric BPAN and demonstrated that susceptibility-weighted imaging and QSM detected progressive iron deposition predominantly within the globus pallidus and substantia nigra during disease progression. In their cohort, iron-related signal abnormalities were not consistently evident on the earliest MRI examinations but became detectable during follow-up, indicating that regional iron accumulation evolves in BPAN. The study also reported persistently increased QSM values in the globus pallidus and substantia nigra compared with controls (Kimura et al., 2021). Collectively, these studies demonstrate that recent NBIA research has begun to integrate cellular and imaging approaches to characterize iron localization at multiple biological levels. Cellular investigations have shown altered intracellular iron homeostasis in ATP13A2-associated disease, whereas quantitative neuroimaging has localized iron predominantly to the globus pallidus and substantia nigra in several NBIA subtypes. These complementary observations provide a more detailed description of iron localization than conventional MRI alone, while also illustrating that the reported patterns depend on the disease subtype and experimental model examined.

## Clinical Course and Emerging Therapies

**Figure 3.**
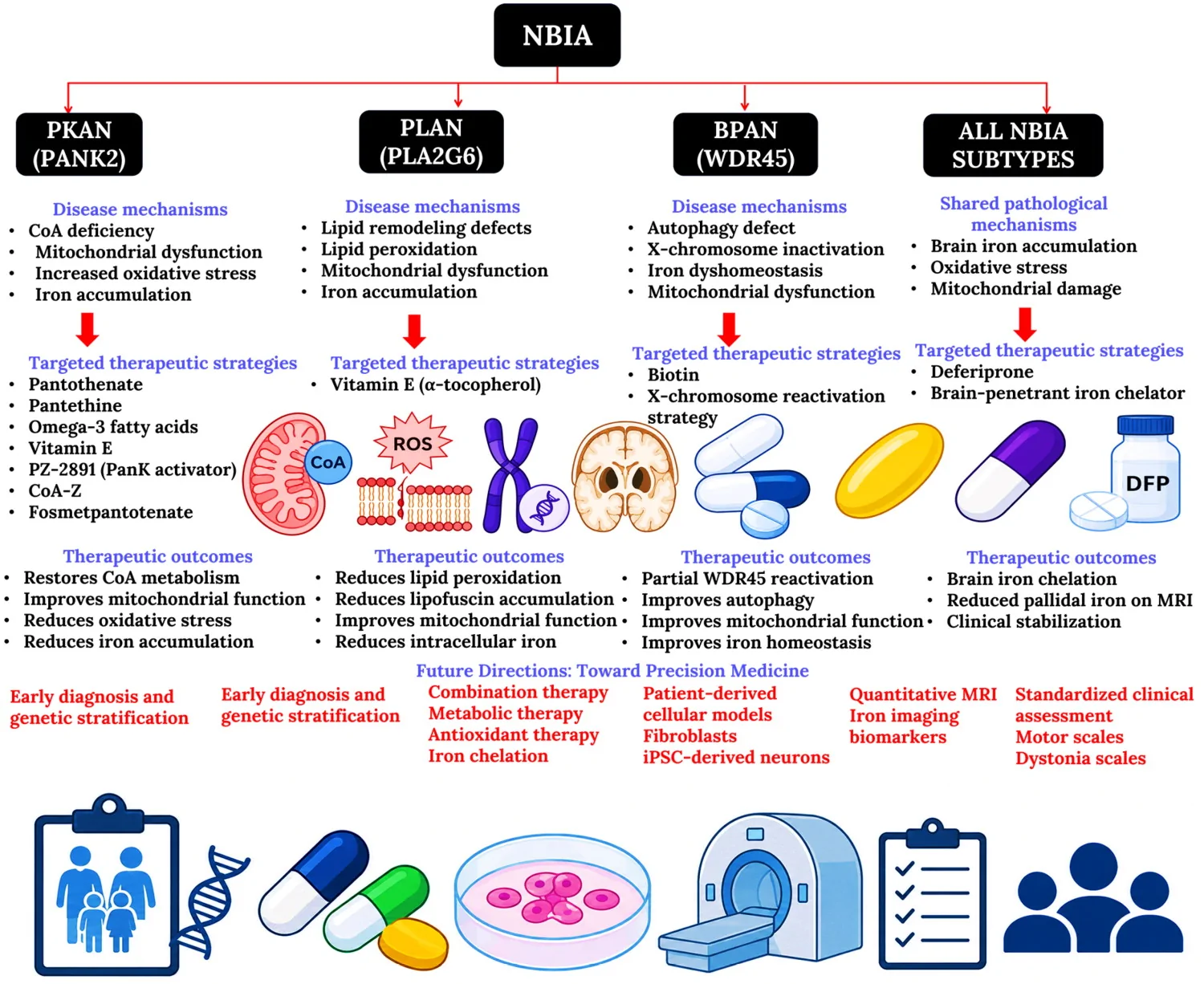
Mechanism-guided therapeutic roadmap for emerging therapies in neurodegeneration with brain iron accumulation (NBIA). Therapeutic strategies targeting major NBIA subtypes are summarized. PKAN therapies aim to restore CoA metabolism and mitochondrial function; PLAN therapies target lipid peroxidation and oxidative stress; BPAN approaches focus on autophagy and X-chromosome reactivation; and deferiprone targets brain iron accumulation across NBIA subtypes. The lower panel highlights future precision medicine approaches, including early genetic diagnosis, combination therapies, patient-derived cellular models, quantitative MRI biomarkers, standardized clinical assessment, and genetically stratified clinical trials.

Pantothenate kinase-associated neurodegeneration (PKAN), PLA2G6-associated neurodegeneration (PLAN), and other NBIA subtypes remain without proven disease-modifying therapies, but recent small clinical and translational studies suggest that multitarget nutritional supplementation, antioxidant strategies, and long-term brain penetrant iron chelation may partially correct cellular abnormalities or stabilize clinical course in selected patients, as highlighted in **Table 7.** Together, these studies illustrate how therapies targeting CoA metabolism and mitochondrial function (PKAN), lipid peroxidation and membrane integrity (PLAN), and non-transferrin-bound iron (deferiprone across NBIA) may intersect mechanistically around oxidative stress, iron dyshomeostasis, and mitochondrial damage, as highlighted in **Figure 3.**

**Table 7.** Therapeutic strategies for neurodegeneration with brain iron accumulation: established approaches and recent advances.

Reference	NBIA subtype	Therapeutic strategy	Main findings
(Iankova et al., 2021)	All NBIA	Artesunate	Promotes TfR1 palmitoylation to reduce cellular iron uptake.
	PKAN	Coenzyme A supplementation	Restores CoA metabolism in preclinical models.
	PKAN	Pantothenate supplementation	Beneficial in models with residual PANK2 activity.
	PKAN	Fosmetpantotenate	Phosphopantothenate replacement therapy.
	PKAN	4′-Phosphopantetheine	CoA precursor therapy.
	PKAN	Pantethine	CoA precursor supplementation.
	PKAN	S-Acetyl-4′-phosphopantetheine	Stable phosphopantetheine analogue.
	PKAN	Pantazines (PZ-2891)	Small-molecule PANK activator.
	PKAN	VTAC compounds	Pharmacological activation of PANK enzymes.
	PKAN	AAV9-mediated gene therapy	PANK2 gene replacement.
	PLAN	Deuterated polyunsaturated fatty acids (D-PUFAs)	Reduces lipid peroxidation.
	PLAN	Desipramine	Targets phospholipid metabolism.
	PLAN	AAV9-hPLA2G6 gene therapy	Gene replacement approach.
	BPAN	Rapamycin	Autophagy induction.
	BPAN	Tauroursodeoxycholic acid (TUDCA)	Reduces ER stress and neuronal injury.
	MPAN	Experimental therapies	Early-stage therapeutic development.
	Aceruloplasminemia	Ceruloplasmin enzyme replacement	Restores ferroxidase activity.
(Sharma et al., 2018)	PKAN	Pantazine (PZ-2891)	BBB-penetrant allosteric PANK activator that increases brain CoA and improves survival in mouse models.
(Bhatnagar et al., 2014)	X-linked disorders (translational relevance to BPAN)	DNMT1 inhibition-mediated inactive X chromosome reactivation	Pharmacological Xi reactivation as an epigenetic therapeutic strategy.
(Carrette et al., 2018)	X-linked disorders (translational relevance to BPAN)	Xist antisense oligonucleotide + DNA methylation inhibitor	Synergistic Xi reactivation strategy restoring expression of the healthy X-linked allele.
(Klopstock et al., 2021)	PKAN	Fosmetpantotenate (FORT trial)	Phase III trial demonstrated acceptable safety but no significant clinical efficacy.
(Reche-López et al., 2025)	BPAN	Biotin-induced X chromosome reactivation	Reactivated the normal WDR45 allele and corrected autophagy, mitochondrial dysfunction, and iron dysregulation.
(Villalón-García et al., 2022)	PLAN	Vitamin E	Reduced lipid peroxidation, iron accumulation, and mitochondrial dysfunction in patient-derived cells.
(Romano et al., 2022)	NBIA	Deferiprone	Reduced brain iron accumulation with favorable long-term safety.
(Álvarez-Córdoba et al., 2022)	PKAN	Nutritional supplementation	Improved mitochondrial function and reduced iron accumulation in responder cells.
(Pereira et al., 2024)	PKAN	Pantothenate + pantethine + omega-3 + vitamin E	Improved cellular abnormalities and stabilized/improved clinical symptoms in a pilot study.
(Nataraj et al., 2023)	MEPAN	Deep brain stimulation	Improved refractory dystonia and motor outcomes.
(Cavestro et al., 2026)	CoPAN	Leriglitazone	Reduced neuroinflammation and improved motor deficits in a preclinical model.

Pereira et al. examined a multitarget nutritional strategy in PKAN, a PANK2-related NBIA subtype characterized by CoA deficiency, mitochondrial dysfunction, iron accumulation, oxidative stress, and progressive dystonia, rigidity, Parkinsonism, dysarthria, and movement disability. They conducted a single-arm, open-label pilot study in three genetically confirmed PKAN patients, combining pantothenate, pantethine, omega-3 fatty acids, and vitamin E with standard neurological medications, while testing the same supplement combination in patient-derived fibroblasts. *In vitro,* supplementation significantly reduced intracellular iron accumulation, increased PANK2 transcript and protein expression, restored mitochondrial acyl carrier protein (mtACP), reduced lipid peroxidation, and partially restored pyruvate dehydrogenase (PDH) activity, indicating improvement in multiple PANK2-linked mitochondrial and iron handling abnormalities. Clinically, patients received the supplements for 24 weeks; treatment was well tolerated, with only mild transient gastrointestinal adverse events and stable liver, renal, and hematological parameters. Two patients showed improvement in motor and dystonic symptoms, while the third remained stable without further deterioration, suggesting that this approach may help improve or stabilize disease course, particularly in individuals with residual PANK2 activity. Pereira et al. concluded that multitarget supplementation directed at CoA metabolism, oxidative stress, mitochondrial dysfunction, and iron dysregulation has potential as augmentation therapy for selected PKAN patients, but emphasized the need for larger, controlled trials to confirm efficacy and durability (Pereira et al., 2024).

Beyond nutritional supplementation strategies in PKAN, several CoA-directed pharmacological approaches have also been explored. In a multicentre, randomized, double-blind, placebo-controlled phase 3 trial (FORT), Klopstock et al. evaluated fosmetpantotenate, a phosphopantothenate precursor intended to bypass the PANK2 block, but the study did not show superiority over placebo on the primary motor scale or secondary clinical endpoints, underscoring the difficulty of translating CoA-replacement concepts into robust clinical benefit in advanced disease (Klopstock et al., 2021). In parallel, Sharma et al. described the development of PZ-2891, a small-molecule “pantazine” that allosterically activates pantothenate kinase, and demonstrated in Pank-deficient mice that chronic oral PZ-2891 increased brain and liver CoA levels while improving body weight, locomotor activity and survival, thereby providing strong preclinical support for PanK activation as a disease-relevant strategy in PKAN (Sharma et al., 2018). Building on these data, Iankova et al. reviewed the emerging therapeutic landscape and highlighted CoA-Z, a 4′-phosphopantetheine-containing compound designed to restore CoA homeostasis in PKAN, which has progressed into phase 2 clinical evaluation (Iankova et al., 2021), together with PZ-2891 and related agents as key candidates in the next generation of PKAN trials.

Villalón García et al. addressed PLA2G6-associated neurodegeneration (PLAN), an autosomal recessive NBIA subtype caused by loss of iPLA2β, in which impaired phospholipid remodeling promotes lipid peroxidation, oxidative stress, mitochondrial dysfunction, and iron accumulation. Using patient-derived fibroblasts and induced neurons generated by direct reprogramming, they showed reduced iPLA2β expression together with increased lipid peroxidation, intracellular iron accumulation, lipofuscin deposition, elevated mitochondrial ROS, loss of mitochondrial membrane potential, fragmented networks, and structural mitochondrial abnormalities, indicating that these cellular models faithfully reproduce major disease features. Experimentally induced lipid peroxidation in healthy fibroblasts increased intracellular iron and lipofuscin, and pharmacological iPLA2β inhibition similarly promoted lipid peroxidation and iron overload, supporting a tight link between lipid peroxidation and iron accumulation in PLAN. Treatment with 50 μM α-tocopherol (vitamin E) markedly reduced lipid peroxidation, intracellular iron, lipofuscin, mitochondrial oxidative stress, and mitochondrial lipid peroxidation, while improving mitochondrial membrane potential, morphology, cell viability, and the enlarged cellular phenotype in PLAN fibroblasts, despite not restoring iPLA2β expression, indicating that benefits stem from antioxidant rather than gene corrective effects. Similar protective effects were seen in PLAN-derived induced neurons, where vitamin E reduced iron accumulation, soma enlargement, superoxide production, lipid peroxidation, and mitochondrial dysfunction. Villalón García et al. thus demonstrated that patient-derived fibroblasts and induced neurons are valuable PLAN models and that vitamin E can effectively correct several major pathological alterations by blocking lipid peroxidation, highlighting oxidative membrane damage as a promising therapeutic target in PLA2G6-related NBIA (Villalón-García et al., 2022).

Romano et al. approached NBIA more broadly by evaluating oral deferiprone (DFP), a blood–brain barrier penetrant iron chelator, in a prospective long-term study of 10 NBIA patients (4 PKAN, 2 PLA2G6-associated NBIA, 1 neuroferritinopathy, 3 idiopathic NBIA) treated with 15 mg/kg twice daily and followed for a mean of 5.5 ± 2.3 years. Serial MRI showed a significant, progressive reduction of iron in the globus pallidus internus, with R2* values falling from 47.6 ± 6.4 to 37.3 ± 5.8 Hz on the left and from 48.4 ± 6.2 to 37.9 ± 6.6 Hz on the right (both p < 0.0001), confirming sustained neuroradiological improvement. Clinically, half the patients remained neurologically stable, while the others had varying degrees of progression, and no clear correlation emerged between reduction in pallidal iron and clinical outcome, suggesting that radiological improvement, especially in advanced disease, does not necessarily translate into measurable functional recovery. Long-term safety was favorable, with no severe hematological toxicity, though seven patients required oral iron supplementation to maintain ferritin >30 μg/L. Given deferiprone’s short plasma half-life, Romano et al. proposed that more continuous chelation, potentially via thrice daily dosing rather than twice daily, might better prevent oxidative damage from non-transferrin bound iron and yield greater clinical benefit while maintaining safety (Romano et al., 2022).

β-propeller protein-associated neurodegeneration (BPAN) provides a distinct example in which the X-linked inheritance pattern itself may be exploitable therapeutically. Sánchez-Alcázar et al. studied BPAN patient-derived cells carrying heterozygous pathogenic variants in WDR45 and showed that these cells exhibit mitochondrial dysfunction, impaired autophagy and disturbed iron homeostasis, and that biotin treatment increased histone biotinylation, reactivated expression of the wild-type WDR45 allele on the inactive X chromosome, and partially corrected these cellular abnormalities (Reche-López et al., 2025). Conceptually related work in other X-linked disorders, including Rett syndrome, has demonstrated that mixed genetic–pharmacological approaches can achieve partial reactivation of the inactive X chromosome in disease-relevant models (Carrette et al., 2018), and that genetic and pharmacological modulation of silencing pathways can reactivate Xi in mammalian cells (Bhatnagar et al., 2014), supporting X-chromosome reactivation as a plausible future precision medicine strategy for BPAN.

Viewed together (Pereira et al., 2024) and (Villalón-García et al., 2022) indicate that targeted antioxidant and metabolic supplementation can act on several convergent NBIA mechanisms, with pantothenate/pantethine plus omega 3 and vitamin E in PKAN aiming to restore CoA biosynthesis, improve mitochondrial phosphopantetheinylation, reduce lipid peroxidation, and attenuate iron overload, while α tocopherol in PLAN primarily blocks lipid peroxidation and stabilizes mitochondrial membranes, thereby reducing iron accumulation, lipofuscin deposition, and mitochondrial oxidative stress and partially normalizing membrane potential and morphology. In both settings, the interventions correct key cellular phenotypes, iron dyshomeostasis, mitochondrial dysfunction, and oxidative damage in patient-derived fibroblasts and induced neurons, and in PKAN, are accompanied by stabilization or modest improvement of motor and dystonic symptoms in small open-label cohorts, particularly where residual PANK2 or PLA2G6 function is present, suggesting that therapeutic window and baseline enzyme activity may critically influence responsiveness (Romano et al., 2022) complements these subtype focused approaches by showing that long term, brain penetrant iron chelation with deferiprone can robustly and progressively reduce pallidal iron across genetically diverse NBIA forms, yet the absence of a consistent relationship between reduced iron burden and clinical improvement, especially in more advanced patients, underscores that iron removal alone may not be sufficient to reverse established neurodegeneration and highlights the likely need for earlier intervention and combination strategies that also address mitochondrial and membrane damage. Important limitations across these studies include very small sample sizes, single-arm or open-label designs without placebo controls, relatively short or moderate clinical follow-up compared with the chronic nature of NBIA, and the lack of randomized trials to define optimal dosing schedules, treatment duration, and genotype or phenotype-specific inclusion criteria, making it difficult to generalize efficacy beyond the reported cases. From a mechanistic and translational standpoint, however, these data support a therapeutic roadmap in which NBIA management increasingly combines subtype specific metabolic and antioxidant interventions (for example, pantothenate/pantethine plus omega 3/vitamin E in PKAN and vitamin E in PLAN) with more general iron targeting approaches such as deferiprone, and in which future studies are conducted in carefully phenotyped, genetically stratified cohorts, using quantitative iron imaging, standardized motor and dystonia scales, and, where feasible, parallel patient derived cellular models to refine dose, identify biological responders, and better link cellular correction to meaningful clinical benefit.

## Challenges and Future Research Directions

Despite substantial advances in understanding neurodegeneration with brain iron accumulation (NBIA), several fundamental challenges continue to impede mechanistic interpretation and therapeutic translation. The evidence synthesized throughout this review indicates that NBIA should no longer be viewed solely as a disorder of iron overload but rather as a heterogeneous group of diseases in which defects in coenzyme A metabolism, mitochondrial bioenergetics, lipid remodeling, and autophagy–lysosomal function converge to produce secondary brain iron accumulation, oxidative stress, and lipid peroxidation, ultimately leading to ferroptotic neuronal death. However, the temporal hierarchy linking these interconnected pathways remains incompletely resolved.

One of the major challenges is determining the sequence of pathogenic events across different NBIA subtypes. Studies of PKAN, CoPAN, and MEPAN consistently demonstrate that mitochondrial dysfunction, defective Fe–S cluster biogenesis, impaired mitochondrial fatty acid synthesis, and altered phosphopantetheinylation frequently precede detectable iron accumulation, supporting the hypothesis that mitochondrial failure is an initiating event (Álvarez-Córdoba et al., 2021; Cavestro et al., 2026; Di Meo et al., 2020; Murdock et al., 2025). In contrast, disorders such as BPAN and PLAN emphasize lysosomal dysfunction, impaired autophagy, defective ferritinophagy, and lipid peroxidation as early pathogenic mechanisms (Álvarez-Córdoba et al., 2021; Diaw et al., 2022; H. E. Lee et al., 2024). Future investigations should therefore employ longitudinal experimental systems capable of defining the temporal relationship among mitochondrial dysfunction, oxidative stress, lipid peroxidation, ferroptosis, and iron accumulation rather than considering these mechanisms independently.

Another important limitation is the incomplete understanding of the subcellular localization and biochemical form of accumulated iron. Increasing evidence suggests that iron is not uniformly distributed but may accumulate within ferritin, lipofuscin, mitochondria, lysosomes, or other intracellular compartments depending on disease subtype and disease stage (Agostini et al., 2024; Dusek et al., 2022). In patient-derived PKAN fibroblasts, iron is partly sequestered within lipofuscin granules, which possess a high iron content and are markedly increased, while impaired mitochondrial iron–sulfur cluster biogenesis is associated with depletion of the cytosolic labile iron pool and compensatory upregulation of iron uptake. These findings indicate that total iron accumulation alone does not accurately reflect iron bioavailability or its contribution to cellular toxicity (Alvarez-Cordoba et al. 2019). Likewise, mitochondrial iron deficiency may coexist with cytosolic iron overload, highlighting profound compartment-specific iron dysregulation (Agostini et al., 2024; Suárez-Carrillo et al., 2023). Future studies combining quantitative susceptibility mapping (QSM), synchrotron-based elemental imaging, spatial transcriptomics, and single-cell metal imaging should define where iron accumulates and how compartment-specific iron pools influence neuronal degeneration.

Although this review incorporates substantially more human cellular studies than previous syntheses, another critical challenge remains the development of physiologically relevant human disease models. Traditional fibroblast cultures and rodent models have provided important mechanistic insights, but cannot fully reproduce selective neuronal vulnerability or human-specific cellular interactions. Recent advances using patient-derived iPSC-derived dopaminergic neurons, astrocytes, oligodendrocytes, and microglia have demonstrated subtype-specific abnormalities in mitochondrial metabolism, iron trafficking, neuroinflammation, lysosomal biology, and ferroptosis (Cozzi et al., 2025; Efendic et al., 2025; Özata et al., 2026; Santambrogio et al., 2024; Tournois et al., 2026). Future research should move beyond monoculture systems toward multicellular human platforms, including neuron–astrocyte, neuron–microglia, and neuron–oligodendrocyte co-culture models, three-dimensional brain organoids, assembloids, and microfluidic “brain-on-chip” technologies that better reproduce the cellular complexity of NBIA.

An additional challenge concerns the remarkable clinical and genetic heterogeneity of NBIA. Although more than twenty disease-associated genes have now been implicated, most mechanistic studies remain focused on PKAN and PLAN, whereas rarer disorders, including CoPAN, MEPAN, FAHN, REPS1-associated NBIA, MT-CO2-associated NBIA, and ATP13A2-related Kufor–Rakeb syndrome, remain comparatively underexplored (Courtois et al., 2024; Erterek et al., 2026; Khan et al., 2025; Murakami et al., 2026). Larger international patient registries, harmonized phenotyping, standardized MRI protocols, and integrated multi-center genomic databases will therefore be essential to establish genotype–phenotype relationships and improve patient stratification.

Another unresolved issue is the contribution of glial cells and neuroimmune signaling to NBIA progression. Emerging evidence indicates that astrocytes actively regulate iron storage, mitochondrial homeostasis, senescence, and ferroptotic susceptibility, while microglia exhibit altered lysosomal trafficking, immune signaling, and inflammatory secretomes (Cozzi et al., 2025; Özata et al., 2026; Santambrogio et al., 2024). These findings suggest that neurodegeneration in NBIA is unlikely to be purely neuron-autonomous. Future investigations should therefore define how neuron–glia interactions influence disease progression and whether modulation of glial iron metabolism or inflammatory signaling represents a viable therapeutic strategy.

Translationally, one of the greatest challenges remains the absence of effective disease-modifying therapies. Current interventions, including iron chelation, antioxidant supplementation, pantothenate-based metabolic therapies, leriglitazone, RT001, CoA-Z, PZ-2891, and deep brain stimulation, have demonstrated encouraging biochemical or symptomatic improvements but remain supported primarily by preclinical studies, small observational cohorts, or early-phase clinical trials (Cavestro et al., 2026; Pereira et al., 2024; Romano et al., 2022). Future clinical trials should incorporate genotype-specific patient stratification, quantitative imaging biomarkers, fluid biomarkers of ferroptosis and neurodegeneration, standardized clinical outcome measures, and parallel patient-derived cellular assays to identify biological responders and monitor therapeutic efficacy.

The increasing availability of transcriptomics, proteomics, metabolomics, lipidomics, spatial omics, and single-cell sequencing offers an unprecedented opportunity to redefine NBIA pathogenesis at molecular resolution. Integrating these multi-omics datasets with advanced human cellular models and longitudinal clinical cohorts will facilitate the identification of subtype-specific biomarkers and therapeutic targets while clarifying common mechanisms shared across genetically distinct forms of NBIA (Cascone et al., 2026; Wiśniewska et al., 2025). Overall, future research should transition from describing isolated molecular defects toward constructing an integrated systems-level model of NBIA in which mitochondrial dysfunction, lipid remodeling, protein aggregation, amyloidogenesis (Basha et al., 2023; Basha et al. 2025; Basha, Mukunda, et al., 2025; Basha et al. 2026), autophagy–lysosomal impairment, ferroptosis, neuroinflammation, neuronal plasticity, nutritional aspects and iron dyshomeostasis in neurodegenerative disorders are investigated as dynamic and interacting processes (Basha et al. 2026; Vijeev, et al., 2026; Devarakonda et al., 2024; Pithakumar et al., 2026). Such an approach is expected to improve mechanistic understanding, enable precision medicine strategies, and accelerate the development of effective disease-modifying therapies for this highly heterogeneous group of neurodegenerative disorders.

## Conclusion

Neurodegeneration with brain iron accumulation (NBIA) represents a genetically diverse yet mechanistically convergent group of disorders in which defects in coenzyme A biosynthesis, mitochondrial bioenergetics, membrane lipid remodeling, autophagy–lysosomal pathways, and ferroptosis collectively disrupt neuronal homeostasis long before extensive neurodegeneration becomes clinically apparent. By integrating recent mechanistic, cellular, imaging, and translational evidence across both common and rare NBIA subtypes, this review supports an emerging disease model in which mitochondrial dysfunction and lipid metabolic failure constitute the primary pathogenic events, whereas abnormal brain iron accumulation acts predominantly as a secondary amplifier of oxidative stress, lipid peroxidation, ferroptotic susceptibility, and progressive neuronal injury rather than the initiating trigger. Importantly, the synthesis of recent studies demonstrates that iron accumulation is highly compartmentalized, involving mitochondria, lysosomes, ferritin, and lipofuscin depending on disease subtype and disease stage, emphasizing that disturbances in intracellular iron distribution may be more biologically relevant than total tissue iron burden alone. The incorporation of patient-derived iPSC-derived neurons, astrocytes, microglia, oligodendrocytes, and advanced human cellular models has substantially expanded our understanding of cell-type-specific mechanisms, revealing that neuronal dysfunction is closely intertwined with glial pathology, neuroinflammatory signaling, impaired organelle crosstalk, and defective iron handling. These human-relevant models, together with advances in quantitative iron imaging, multi-omics technologies, and functional biomarkers, are reshaping the mechanistic framework of NBIA and providing more predictive platforms for therapeutic discovery. At the translational level, emerging interventions targeting CoA metabolism, mitochondrial function, lipid peroxidation, ferroptosis, neuroinflammation, and iron homeostasis, including metabolic supplementation, antioxidant therapy, iron chelation, and pathway-directed compounds, highlight a transition from purely symptomatic management toward mechanism-based precision medicine, although robust validation through genotype-stratified, multicenter clinical trials remains essential. Overall, the collective evidence reviewed here indicates that future progress in NBIA will depend on integrating advanced human disease models, systems-level molecular profiling, quantitative imaging biomarkers, and precision therapeutic strategies to define disease-specific pathogenic cascades, identify early biomarkers, and develop effective disease-modifying treatments. Such an integrated framework not only refines the current understanding of NBIA pathogenesis but also establishes a foundation for translating mechanistic discoveries into personalized therapeutic interventions for these devastating neurodegenerative disorders.

## Data Availability

Data sharing is not applicable to this article because no new data were created or analyzed in this study.
